# Tundra Soil Viruses Mediate Responses of Microbial Communities to Climate Warming

**DOI:** 10.1128/mbio.03009-22

**Published:** 2023-02-14

**Authors:** Mengzhi Ji, Xiangyu Fan, Carolyn R. Cornell, Ya Zhang, Mengting Maggie Yuan, Zhen Tian, Kaili Sun, Rongfeng Gao, Yang Liu, Jizhong Zhou

**Affiliations:** a School of Biological Science and Technology, University of Jinan, Jinan, Shandong Province, China; b Institute of Marine Science and Technology, Shandong University, Qingdao, Shandong Province, China; c Institute for Environmental Genomics and Department of Microbiology and Plant Biology, University of Oklahoma, Norman, Oklahoma, USA; d Department of Environmental Science, Policy, and Management, University of California, Berkeley, California, USA; e School of Civil Engineering and Environmental Sciences, University of Oklahoma, Norman, Oklahoma, USA; f School of Computer Sciences, University of Oklahoma, Norman, Oklahoma, USA; g Earth and Environmental Sciences, Lawrence Berkeley National Laboratory, Berkeley, California, USA; The University of British Columbia; University of California—Irvine

**Keywords:** climate warming, environmental factors, glycoside hydrolases, viral lifestyle, virus-microbe linkage, virus-host linkage

## Abstract

The rise of global temperature causes the degradation of the substantial reserves of carbon (C) stored in tundra soils, in which microbial processes play critical roles. Viruses are known to influence the soil C cycle by encoding auxiliary metabolic genes and infecting key microorganisms, but their regulation of microbial communities under climate warming remains unexplored. In this study, we evaluated the responses of viral communities for about 5 years of experimental warming at two depths (15 to 25 cm and 45 to 55 cm) in the Alaskan permafrost region. Our results showed that the viral community and functional gene composition and abundances (including viral functional genes related to replication, structure, infection, and lysis) were significantly influenced by environmental conditions such as total nitrogen (N), total C, and soil thawing duration. Although long-term warming did not impact the viral community composition at the two depths, some glycoside hydrolases encoded by viruses were more abundant at both depths of the warmed plots. With the continuous reduction of total C, viruses may alleviate methane release by altering infection strategies on methanogens. Importantly, viruses can adopt lysogenic and lytic lifestyles to manipulate microbial communities at different soil depths, respectively, which could be one of the major factors causing the differences in microbial responses to warming. This study provides a new ecological perspective on how viruses regulate the responses of microbes to warming at community and functional scales.

## INTRODUCTION

Northern-latitude tundra soils store a considerable amount of C (estimated to be 1,672 to 1,832 Pg), accounting for about half of the global soil organic carbon (SOC) reservoir (1–3). Historical record shows that the climate warming-related temperature increase is more rapid in northern-latitude areas than the global average (2). The sustained temperature rise causes the highly vulnerable permafrost to thaw, stimulating the microbial degradation of SOC previously restricted by low temperature and freezing conditions (4). This may eventually lead to large amounts of soil C being converted to greenhouse gases (mainly CO_2_ and CH_4_) and discharged into the atmosphere, further exacerbating climate warming (5).

Various studies from past decades have a provided mechanistic understanding of microbial community responses to warming in permafrost regions (6–8). As part of a unique effort focusing on relatively well-drained upland tundra undergoing recent permafrost degradation, the Carbon in Permafrost Experimental Heating Research (CiPEHR) site was established in central Alaska in 2008 (2, 9). At the CiPEHR site, the Alaskan tundra soils were subjected to *in situ* experimental warming (~1.1°C above ambient temperature averaged across five winters) (2). Previous studies found that short-term (~1.5 years) climate warming did not significantly affect the taxonomic composition of microorganisms but did significantly alter community functional structure based on GeoChip analysis (4). Under continuous warming (~5 years), the functional traits and taxonomic composition of bacterial communities changed significantly, along with increased abundances of C decomposition and methanogenesis genes (2, 10). Meanwhile, microbial responses to warming also varies at different soil depths of permafrost region (2). In addition, the fungal community showed an enhancement in C degradation capacity and a shift in functional composition under winter warming (11). However, most of the current studies have focused only on prokaryotes and lower eukaryotes, but the responses of the viral community to climate warming have received little attention.

As an essential component of the ecological community, viruses are considered to be the center of ecological interactions in aquatic systems, influencing microbial dynamics through viral lysis, regulating microbial physiology through temperate infections and gene transfer between microbes, and directly impact biogeochemical cycles in the ocean by encoding auxiliary metabolic genes (AMGs) (12–14). Although the role of viruses in tundra soil systems remains understudied, the Carbohydrate-Active enZymes (CAZymes) and other AMGs with the functions of C utilization encoded by tundra soil viruses have been confirmed to be crucial in the soil C cycle (15, 16). In addition, it has been discovered in recent studies that viruses can regulate the process of C cycling by actively infecting organisms that are critical to the soil C cycle under subfreezing anoxic conditions, highlighting the modulatory role of viruses as a major community-structuring agent on tundra soil C loss (15, 16). Thus, changes in AMGs encoded by viruses and virus-microbe interaction may also be crucial for the response of the entire tundra soil microbial system to climate warming.

Here, we performed a *de novo* assembly of data obtained from shotgun metagenomic sequencing of northern-latitude permafrost region (2) to mine and analyze the viral information in different depths and temperatures and conducted a GeoChip analysis of viral genes in this region (17). The main objectives of this study were (i) to determine the changes in soil viral community and functional gene structures after ~5 years of experimental warming, (ii) to investigate how viral communities and functions are shaped by soil depth and other environmental factors, (iii) to explore whether viral effects are important factors causing the different feedbacks of microbial communities to warming, and (iv) to analyze virus-microbe linkages to gain a comprehensive understanding of the biogeochemical role played by viruses in tundra soil ecosystems. Our results indicated that viruses adopted different strategies to regulate microbial communities at different depths, which can cause different microbial responses to warming.

## RESULTS

### Recovery and analysis of viruses from different samples.

**FIG 1:**
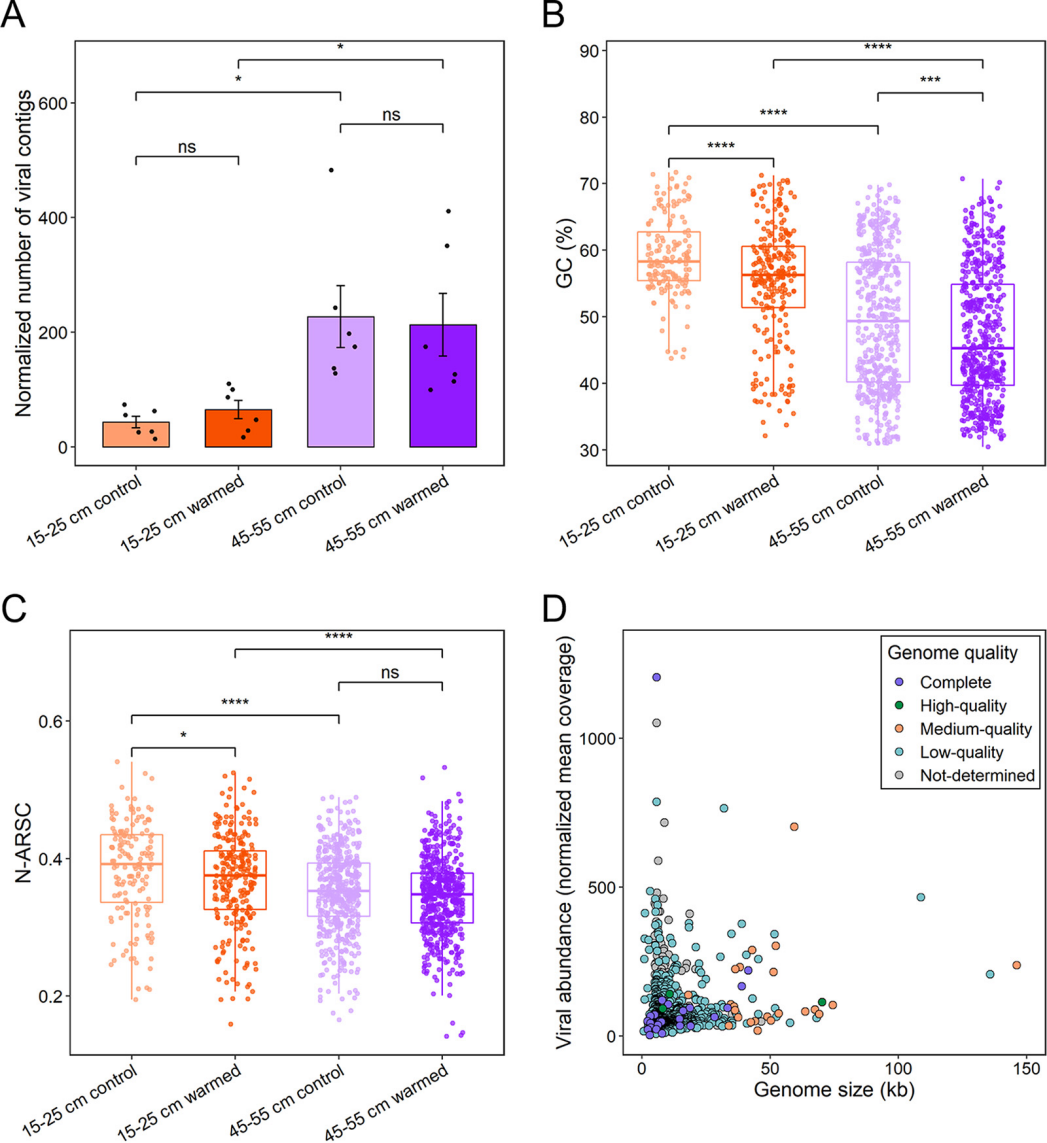
Quality and characterization statistics of viral genomes. (A) Normalized number of viral genomes (dereplicated within each data set) recovered from the four data sets. The dereplicated mVCs within each data set are normalized based on the reads number of each sample. Significant differences were determined using a Student *t* test (ns, no significant difference; *, *P < *0.05). (B) GC content of viral genomes in different data sets. The GC content significance was determined using a Student *t* test (***, *P < *0.001; ****, *P < *0.0001). (C) Nitrogen atoms per residue side chain (N-ARSC) analysis of viral genomes in different data sets. The significance of N-ARSC was determined using a Student *t* test (ns, no significant difference; *, *P < *0.05; ****, *P < *0.0001). (D) Relationship between the length of viral genomes (*x* axis) and their normalized abundances in data sets (*y* axis).

The number of recovered viruses was related to the soil depth rather than the temperature. With the same sequencing effort, more metagenomic viral contigs (mVCs) were recovered at 45 to 55 cm than at 15 to 25 cm (Student *t* test, *P < *0.05) (Fig. 1A). However, it appeared that experimental warming had no significant effect on the number of mVCs recovered from this permafrost region at the two depths (Fig. 1A).

Soil depth and warming were critical in affecting the composition and structure of viral genomes, but depth could be more important. First, the GC contents of mVCs across different data sets were compared, and the results showed that the GC contents of mVCs at 45 to 55 cm was significantly lower than that at 15 to 25 cm (Student *t* test, *P < *0.001) (Fig. 1B). In addition, the GC contents of mVCs at both depths was significantly reduced with experimental warming (Student *t* test, *P < *0.001) (Fig. 1B). Linear mixed-effects model analysis showed that soil depths (*R*^2^ = 0.1395, *P < *0.001) had a greater effect on the GC contents of mVCs than warming (*R*^2^ = 0.0272, *P < *0.001) (Fig. 1B). Since the N atoms per residue side chain (N-ARSC) of microbial genomes are usually driven by the surrounding environmental energy and nutrient (18, 19), we next analyzed the N-ARSC of recovered mVCs. The results showed that the N-ARSC of mVCs was significantly higher (Student *t* test, *P < *0.001) at 15 to 25 cm than at 45 to 55 cm (Fig. 1C). The higher N content at 15 to 25 cm than at 45 to 55 cm (Student *t* test, *P < *0.001) indicated that the features of viral genomes might be driven by the surrounding N availability (2, 19). Experimental warming resulted in a reduction in the N-ARSC of mVCs at 15 to 25 cm and 45 to 55 cm, but only significantly (Student *t* test, *P < *0.05) at 15 to 25 cm (Fig. 1C). Further linear mixed-effects model analysis showed that soil depths (*R*^2^ = 0.0319, *P < *0.001) also had a greater effect on the N-ARSC of mVCs than warming (*R*^2^ = 0.0068, *P < *0.01) (Fig. 1C).

### Drivers of viral community and functional gene composition.

**FIG 2:**
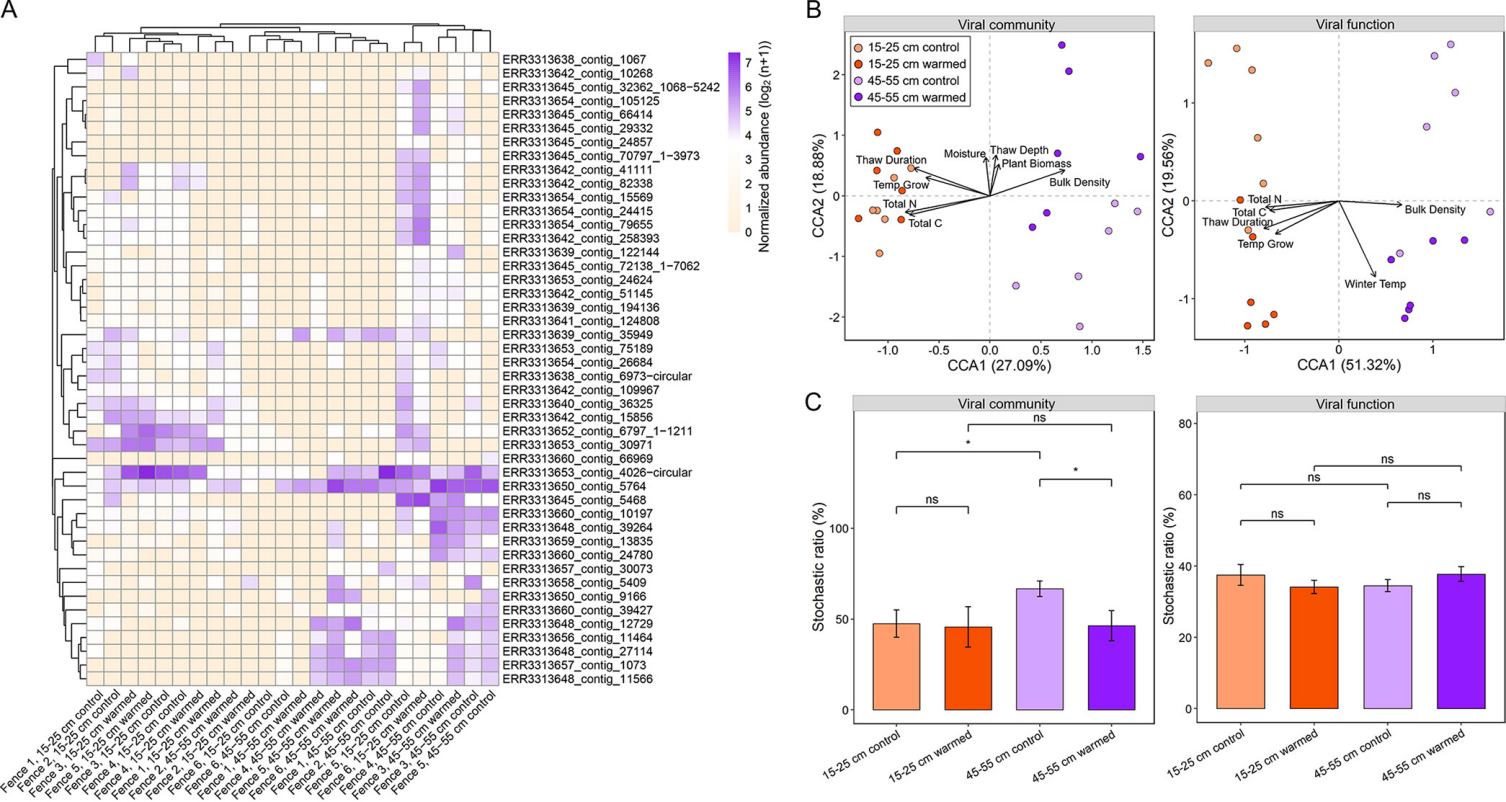
Analysis of viral communities and functions across different data sets. (A) Heatmap showing the abundance patterns of 46 vOTUs that were detected at all temperatures and depths. The color intensity in each panel represents the normalized abundance of each vOTU. The vOTUs are clustered based on the Bray-Curtis dissimilarity matrix. (B) Canonical correspondence analysis (CCA) of viral community (or viral function) and environmental factors. The values in axes 1 and 2 are percentages of variations in the viral community (or viral function) that the axis can explain and the relationship is significant (*P < *0.05). (C) Stochastic ratio of viral community or function for warming and control groups in each depth. The bars represent the mean values of stochastic ratios, and the error bars represent the standard errors. Significant differences were determined using a Student *t* test (ns, no significant difference; *, *P < *0.05).

After dereplication of all recovered viral genome sequences, 1,385 species-level viral operational taxonomic units (vOTUs) were used for downstream analyses. vOTU rarefaction curve analysis showed that the amount of sequencing data appeared to be sufficient for detecting dominant viral members in the permafrost soil (see Fig. S1 in the supplemental material). The completeness of viral representative genomes in the 1,385 vOTUs was as follows: 2% complete genomes and/or high quality, 2% medium quality, 71.6% low quality, and others were not determined (Fig. 1D). Next, we analyzed the abundance patterns of the vOTUs across different depths and temperatures. The 1,385 vOTUs were clustered across different samples based on Bray-Curtis distances, forming two main groups corresponding to different soil depths (see Fig. S2A). Only 46 of the 1,385 vOTUs were detected at all temperatures and depths (see Fig. S2B). Most of these vOTUs were clustered and had closer normalized abundance within the same soil depth (Fig. 2A). Experimental warming had no significant effect on the abundance patterns of vOTUs (Fig. 2A).

10.1128/mbio.03009-22.1 FIG S1 vOTU accumulation curves for the viral data sets in this permafrost region. The black lines within the box indicate the median value of the vOTU number, and the ranges of the error bars represent random replicates. Download FIG S1, TIF file, 0.9 MB. Copyright © 2023 Ji et al. 2023 Ji et al. https://creativecommons.org/licenses/by/4.0/ This content is distributed under the terms of the Creative Commons Attribution 4.0 International license.

10.1128/mbio.03009-22.2 FIG S2 Analysis of viral diversity and abundance across different data sets. (A) Heatmap showing the abundance patterns of 1,385 vOTUs. Samples and vOTUs are clustered based on the Bray-Curtis dissimilarity matrix. (B) Number of vOTUs with abundance associations in the four data sets. (C to E) Shannon diversity, Simpson index, and richness indicate the diversity and richness of viral community across different data sets. A Student *t* test was used to determine the significance. ns, no significant difference (*P > *0.05). (F) NMDS analysis of viral communities based on the Bray-Curtis dissimilarity matrix calculated by the normalized mean coverage of vOTUs. ANOSIM was applied to detect the differences of viral communities between different depths or temperatures. Download FIG S2, TIF file, 1.3 MB. Copyright © 2023 Ji et al. 2023 Ji et al. https://creativecommons.org/licenses/by/4.0/ This content is distributed under the terms of the Creative Commons Attribution 4.0 International license.

Multiple analyses revealed that soil depth rather than warming was the main factor influencing the distribution of viral communities in this permafrost region. First, the composition and diversity of viral communities in different depths or temperatures were analyzed based on the normalized abundance of vOTUs. The Shannon diversity, richness, and Simpson index indicated that neither warming nor depth had a significant effect on the diversity of viral communities (see Fig. S2C to E). Further analysis using nonmetric multidimensional scaling (NMDS) showed that viral communities were clustered according to soil depth (see Fig. S2F). Analysis of similarity (ANOSIM) indicated that viral compositions of warmed plots (*P = *0.002) and control plots (*P = *0.0032) were significantly different at the two soil depths (see Fig. S2F). Experimental warming affected viral composition at neither 15 to 25 cm (*P = *0.8236) nor 45 to 55 cm (*P = *0.5171) (see Fig. S2F). The composition of viral communities was significantly correlated with environmental factors (Mantel test, *R *=* *0.6522, *P = *0.001). A significant canonical correspondence analysis (CCA) model (permutation test, *P < *0.001) showed that total N, total C, soil thaw duration, growing season temperature, moisture, soil bulk density, plant biomass, and soil thawing depth were important environmental factors significantly controlling the viral community structure (permutation test, *P < *0.05) (Fig. 2B). Variation partitioning analysis (VPA) showed that the eight environmental factors explained 42.9% of the variation in viral communities, suggesting that they were important drivers shaping viral community composition (Fig. 2B).

In contrast to shotgun sequencing, microarray-based hybridization is able to provide more quantitative information (20, 21). Thus, these samples were also analyzed by functional gene arrays. GeoChip 5.0 analysis identified a total of 127 DNA viral genes of 2,848 virus-specific probes (17, 22) at the two depths of this permafrost region. Among these, 69 genes were related to prokaryotic viruses and 58 genes were associated with eukaryotic viruses. Among them, the majority of viral genes detected were related to replication (59 of 127) and viral structure (52 of 127), which could be mainly because the viral probes on the GeoChip were most derived from these genes. In addition, some viral genes had functions related to infection (2 of 127) and lysis (14 of 127) were also detected. Experimental warming had no significant effect on viral functional gene structures based on GeoChip at neither 15 to 25 cm (ANOSIM, *P = *0.0841) nor 45 to 55 cm (ANOSIM, *P = *0.0949) (Fig. 2B). Consistent with community composition, soil depth had a significant effect (ANOSIM, *P = *0.0001) on viral functional gene structures based on GeoChip (Fig. 2B). In addition, the function of viruses was significantly correlated with environmental factors (Mantel test, *R *=* *0.391, *P = *0.001). The CCA model (permutation test, *P < *0.001) and VPA showed that six significant environmental factors, including total N, total C, growing season temperature, soil bulk density, soil thaw duration, and winter temperature (permutation test, *P < *0.001), explained 49.8% of the variation in viral functional composition, suggesting that environmental factors were also important drivers shaping viral function (Fig. 2B).

Null model analysis was then employed to discern the relative importance of deterministic and stochastic processes in driving viral community and functional gene structures. For viral communities, only the stochastic ratio of 45 to 55 cm control was >50%, suggesting that deterministic processes played more important roles in driving viral communities in the permafrost region (Fig. 2C). Interestingly, warming significantly decreased (Student *t* test, *P < *0.05) the stochastic ratio of viral communities at 45 to 55 cm (Fig. 2C), suggesting that warming could impose deterministic effects on viruses (23). For viral function, stochastic ratios were consistently <50%, indicating that viral functional traits were highly deterministic (Fig. 2C). Neither warming nor soil depths had significant effects on the stochastic ratios of viral functions (Fig. 2C).

### Taxonomy and clustering of viral community.

**FIG 3:**
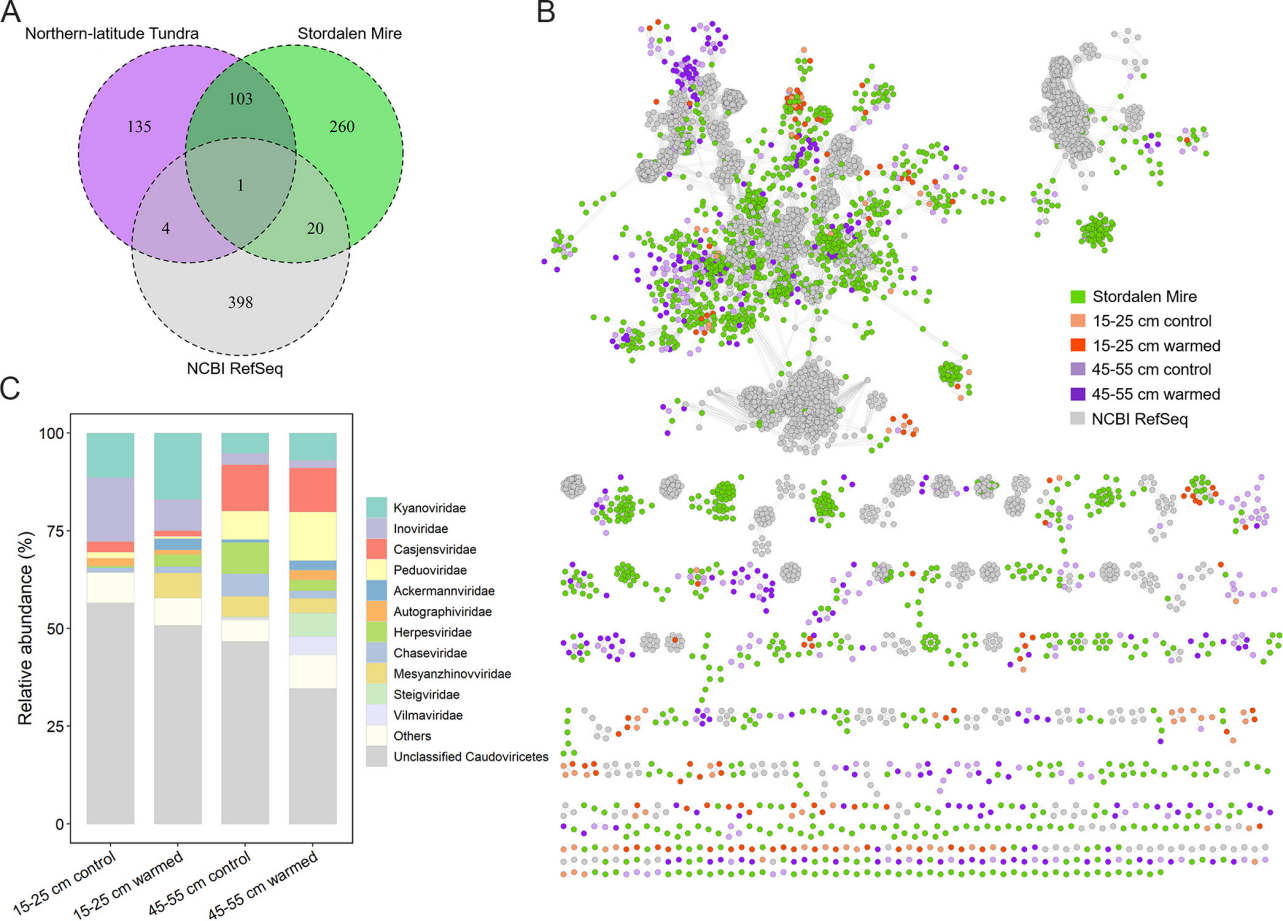
Taxonomy and clustering of tundra viruses with known viral databases. (A) The number of shared viral clusters in Northern-latitude tundra with Stordalen Mire and Viral RefSeq. (B) Network of viral shared protein clusters among Northern-latitude tundra (*n* = 1,385), Stordalen Mire (*n* = 1,907), and Viral RefSeq (*n* = 2,304). The nodes (circular) represent different viral genomes and the edges represent shared protein cluster content. (C) Relative abundances of classified viral taxa (*n* = 171) across different data sets based on the normalized mean coverage. Among them, viral taxa with low relative abundance (<2%) are classified as “others.”

To determine the similarity of tundra soil viruses, 1,385 vOTUs identified in this study were compared to several publicly available viral sequence data sets: (i) 2,213 bacterial viruses and 91 archaeal viruses (RefSeq v85) (24) and (ii) 1,907 vOTUs, recovered from metagenomes and viromes from Stordalen Mire bulk soil, a long-term climate change research site in northern Sweden (15). In the network of shared protein clusters constructed using previous method (15), 476 (~34%) of 1,385 vOTUs clustered with the publicly available viral data sets, yielding a total of 243 viral clusters (Fig. 3A). However, only five vOTUs were clustered with known viral genomes belonging to five different viral clusters with the majority of the viruses in tundra soil ecosystem remaining unknown (Fig. 3A). Approximately 42.8% (104 of 243) of the viral clusters contained vOTUs from both northern-latitude tundra and Stordalen Mire (Fig. 3A). These vOTUs clustered together in the network, indicating that viral communities in tundra soil from different regions were related, suggesting a broad global geographic distribution of soil viral communities as previously reported (Fig. 3A and B) (15).

According to the taxonomic results of PhaGCN2 based on the latest ICTV classification (https://doi.org/10.5281/zenodo.7442695), 171 (~12.3%) of 1,385 vOTUs were annotated and 105 of 171 (~61.4%) could be further classified at family level. Another 66 of 171 vOTUs could only be classified at the subfamily or genus level, so we assumed that they belong to an unclassified family. Also, 148 of 171 vOTUs were assigned to the class *Caudoviricetes* (mainly including the families *Kyanoviridae*, *Casjensviridae*, and *Peduoviridae*). In addition, 23 vOTUs assigned to other classes potentially belong to the families *Phycodnaviridae*, *Microviridae*, *Tectiviridae*, *Adenoviridae*, *Herpesviridae*, *Lipothrixviridae*, and *Inoviridae* (Fig. 3C). In general, viral taxonomic abundance had relatively similar distribution patterns in the same depth, in particular the viral taxa with high abundance (Fig. 3C). Among them, the relative abundance of *Kyanoviridae* and *Inoviridae* dominated at 15 to 25 cm, while the relative abundance of *Casjensviridae* and *Peduoviridae* dominated at 45 to 55 cm (Student *t* test, *P < *0.05) (Fig. 3C). Experimental warming had no significant effects on the abundance of viral taxa (Fig. 3C). The relative abundances of all viral taxa at family level of two depths did not change significantly under experimental warming (Student *t* test, *P > *0.05) (Fig. 3C).

### Auxiliary metabolic genes harbored in viruses.

**FIG 4:**
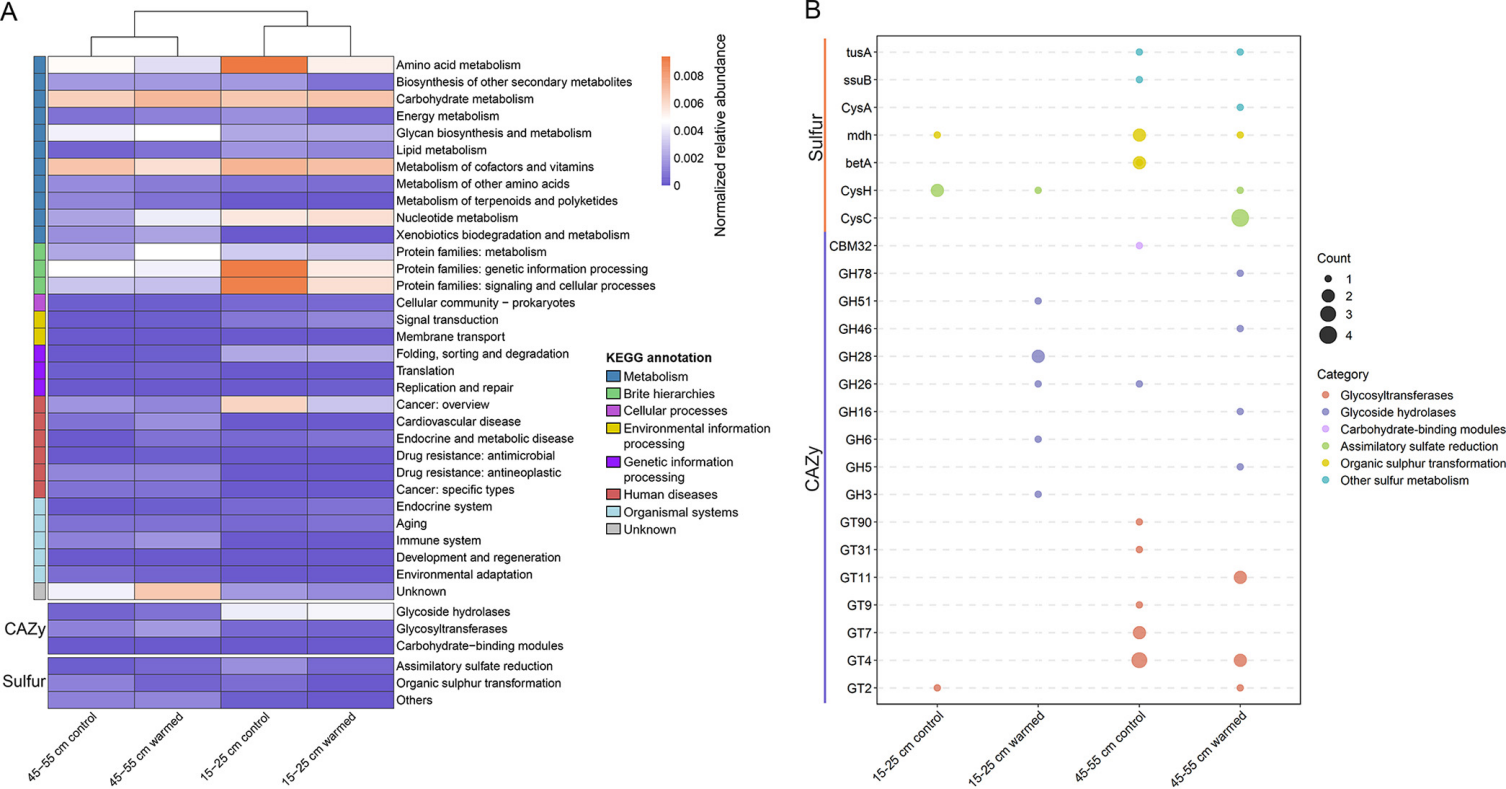
Auxiliary metabolic genes (AMGs) encoded by viruses across different data sets. (A) Heatmap showing the abundance patterns of different types of AMGs encoded by viruses in different groups. Groups were clustered based on the Bray-Curtis dissimilarity matrix. The color intensity in each panel represents the relative abundance of each AMG. (B) Numbers of AMGs related to key C and S cycles carried by viruses in different data sets. The size of bubbles represents the number of annotated viral ORFs, and the different colors represent different categories.

In different ecological environments, viruses can participate directly in biogeochemical cycles through the encoding of AMGs (25, 26). To further investigate the impact of viral communities on soil biogeochemical cycling, we examined and refined 311 AMGs encoded by the vOTUs. In general, tundra soil viruses tended to encode AMGs for “metabolism of cofactors and vitamins” (25.08%) and “carbohydrate metabolism” (22.19%) based on VIBRANT and DRAM-v annotations (see Table S1 in the supplemental material). In addition, AMGs for “nucleotide metabolism” (9.97%), “glycan biosynthesis and metabolism” (13.5%), and “amino acid metabolism” (14.15%) accounted for a large proportion (see Table S1). Based on the normalized relative abundance of AMGs (18, 27), heatmap analysis showed a noticeable depth-stratified distribution of AMGs, which was consistent with previous findings in marine, sulfidic mine tailings, and other environments (Fig. 4A) (18, 28). There was generally no significant effect of warming on the relative abundance of viral AMGs, only the relative abundance of AMGs for “protein families: metabolism” was significantly increased at 45 to 55 cm (Student *t* test, *P < *0.05) (Fig. 4A).

10.1128/mbio.03009-22.8 TABLE S1 Auxiliary metabolic gene information for viral ORFs in the permafrost region. Download Table S1, XLSX file, 0.03 MB. Copyright © 2023 Ji et al. 2023 Ji et al. https://creativecommons.org/licenses/by/4.0/ This content is distributed under the terms of the Creative Commons Attribution 4.0 International license.

Among the viral AMGs related to carbohydrate metabolism, 21 AMGs were annotated as 17 CAZymes, belonging to three CAZymes functional classes (carbohydrate-binding modules, glycoside hydrolases [GHs], and glycosyltransferases) (Fig. 4B; see also Table S1). The detailed functional annotation of these GHs showed that there were a variety of key enzymes with degradation functions for complex polysaccharides in permafrost, mainly including β-glucosidase (GH3), cellulase (GH5), β-glucanase (GH16), β-mannosidase (GH26), chitosanase (GH46), α-l-arabinofuranosidase (GH51), and α-l-rhamnosidase (GH78) (see Table S1). In particular, 10 of 11 GHs were found in the viral genomes of the warmed group, indicating that warming might promote the horizontal gene transfer (HGT) of GHs mediated by viruses (Fig. 4B). However, warming did not cause significant changes in the relative abundance of GHs carried by viruses, but the relative abundance of GHs was significantly greater at depths of 15 to 25 cm than at depths of 45 to 55 cm (Student *t* test, *P < *0.05). Genomic analysis of vOTUs encoding GHs revealed that many vOTUs (6 of 10 vOTUs) were lysogenic viruses containing integrase genes, and all GHs were not previously found in viral genomes (see Fig. S3A). Among them, a high-quality viral genome named ERR3313649_contig_1027 (70 kb, 91.3% completeness) encoding GH16 was identified at the 45 to 55 cm under warming conditions containing several common viral structural proteins (substrate proteins, tail proteins, and terminase large subunit) and a lysogenic profile (integrase and some DNA metabolic modules), and it has abundance only at 45 to 55 cm under warming conditions (see Fig. S3A).

10.1128/mbio.03009-22.3 FIG S3 Genome maps of some AMG-containing contigs and structure models of these AMGs. (A) Arrows represent the locations and directions of predicted genes in the viral genomes. Genes with different functions are annotated by different colors. For CAZymes, genes that can be compared to known viruses are indicated in dark purple; otherwise, they are indicated in light purple. (B) S metabolism-related AMGs that can be compared to known viruses are indicated in dark green; otherwise, they are indicated in light green. (C) Protein structures of selected AMGs based on structural modeling using Phyre2. Download FIG S3, TIF file, 2.4 MB. Copyright © 2023 Ji et al. 2023 Ji et al. https://creativecommons.org/licenses/by/4.0/ This content is distributed under the terms of the Creative Commons Attribution 4.0 International license.

Markedly, sulfur (S) metabolism-related AMGs carried by viruses were also abundant in permafrost (Fig. 4A and B). They were mainly involved in the functions of assimilatory sulfate reduction or organic S transformation (Fig. 4B). Assimilatory sulfate reduction-related AMGs carried by eight vOTUs were annotated as phosphoadenosine phosphosulfate reductase (*cysH*) and adenylylsulfate kinase (*cysC*) that have been previously identified in a variety of environments (see Fig. S3B and C) (28–30). Genomic analysis showed that all assimilatory sulfate reduction-related AMGs were previously detected in the viral genomes (31, 32), and five of them were present at 45 to 55 cm under warming conditions (Fig. 4B; see also Fig. S3B). In addition, some S metabolism-related AMGs of unknown functions were found at 45 to 55 cm, and the relative abundance of them was significantly higher than at 15 to 25 cm (Student *t* test, *P < *0.05) (Fig. 4B).

### Analysis of viral lifestyles and specific genes in tundra soil.

**FIG 5:**
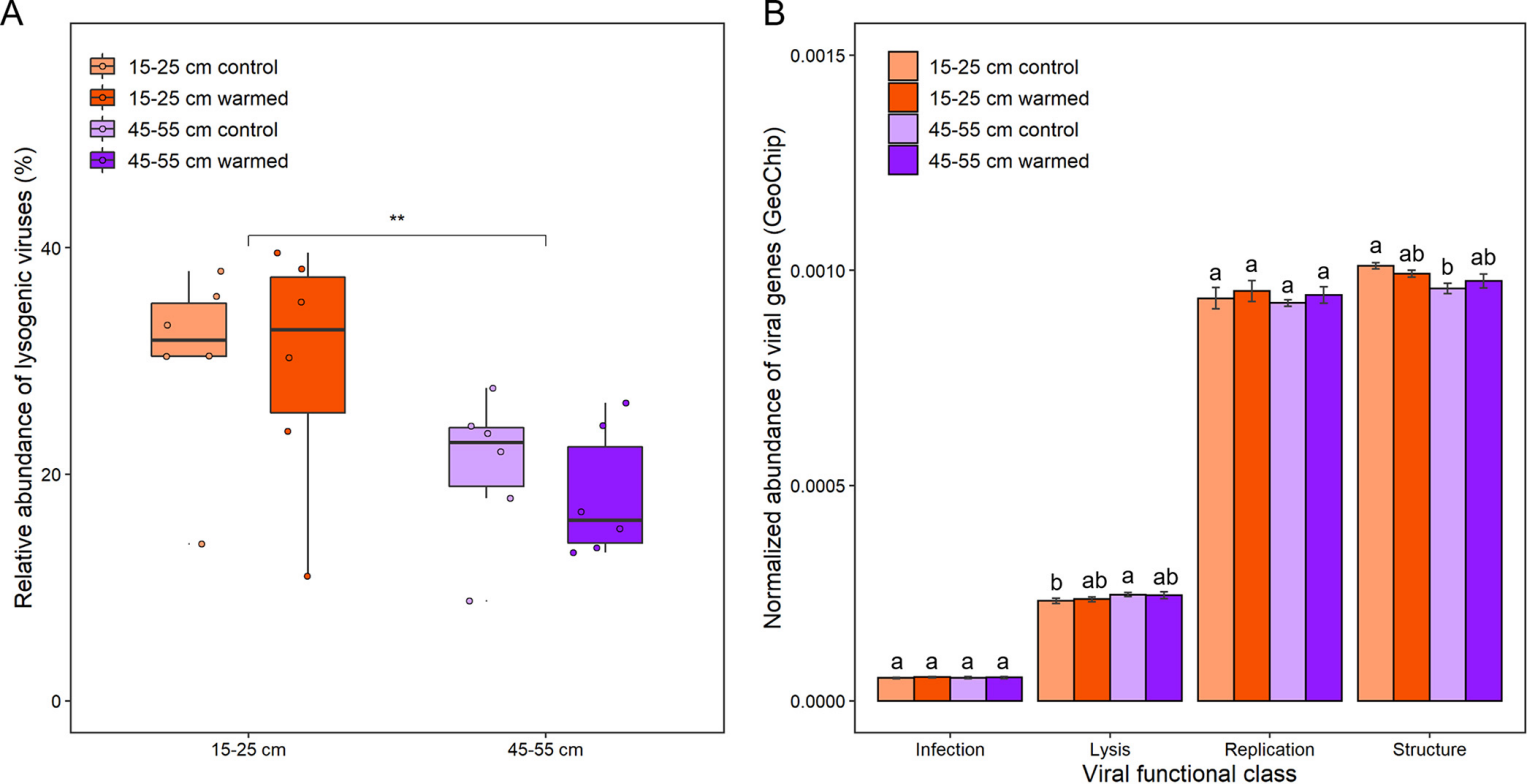
Viral lifestyle and GeoChip genes analysis. (A) Analysis of the relative abundance of lysogenic viruses across different data sets. Significance was determined using a paired *t* test (**, *P < *0.01). (B) Normalized abundances of viral genes assigned to each viral function class in different groups. Error bars represent the means ± the standard errors of the mean (*n* = 6) for the normalized abundances of each viral function class. Lowercase letters (i.e., a, b, and ab) above the error bars show the results of a paired *t* test to determine the significance.

Lysis-lysogeny decision-making in viruses is a critical factor influencing soil nutrient cycling (33). Therefore, the elaboration of viral lifestyles is important in the study of viral ecology (34). In all, 235 lysogenic vOTUs were identified in this study, containing 76 proviruses (integrated lysogenic viruses) and 159 temperate viruses (free lysogenic viruses) (see Fig. S4A). The relative abundance of viruses showed that 5 years of warming did not alter the proportion of lysogenic viruses and lytic viruses (Fig. 5A; see also Fig. S4B). However, the relative abundances of lysogenic viruses at 15 to 25 cm were significantly higher than at 45 to 55 cm (Student *t* test, *P < *0.01), reflecting the difference of viral lifestyle at different depths (Fig. 5A). Markedly, the relative abundances of lysogenic viruses and genes were both correlated positively (*P < *0.05) with total C and N, indicating that eutrophic drove more viruses to adopt lysogenic lifestyles compared to oligotrophic conditions (see Fig. S5A and B).

10.1128/mbio.03009-22.4 FIG S4 Analysis of the lifestyle of tundra soil viruses. (A) Proportions of the number of viruses with different lifestyles across different data sets, including 15 to 25 cm (control, *n* = 141), 15 to 25 cm (warmed, *n* = 227), 45 to 55 cm (control, *n* = 486), and 45 to 55 cm (warmed, *n* = 531). (B) Relative abundances of viruses with different lifestyles across different data sets. Download FIG S4, TIF file, 0.8 MB. Copyright © 2023 Ji et al. 2023 Ji et al. https://creativecommons.org/licenses/by/4.0/ This content is distributed under the terms of the Creative Commons Attribution 4.0 International license.

10.1128/mbio.03009-22.5 FIG S5 Correlation analysis of lysogenic viruses and genes with surrounding nutrients. (A) Correlation analysis between the relative abundance (normalized abundance of lysogenic viruses/total normalized abundance) of lysogenic viruses and total C and N. (B) Correlation analysis between the relative abundance of lysogenic gene (normalized abundance of lysogenic gene/total normalized abundance) and total C and N. Download FIG S5, TIF file, 0.6 MB. Copyright © 2023 Ji et al. 2023 Ji et al. https://creativecommons.org/licenses/by/4.0/ This content is distributed under the terms of the Creative Commons Attribution 4.0 International license.

In addition to identifying lysogenic signals in shotgun sequencing, viral functional genes based on GeoChip are also important information for analyzing viral lifestyles (22). Among the viral genes detected by GeoChip 5.0 (17, 22), the abundance of viral genes related to lysis function was significantly higher at 45 to 55 cm than at 15 to 25 cm (paired *t* test, *P < *0.05), suggesting that some viruses may tend to select for carrying more lysis genes at 45 to 55 cm (Fig. 5B). It should be noted that the lysis-lysogeny decision analyzed by GeoChip could only represent a fraction of tundra viral members, since GeoChip targeted limited classes of viral lysis genes (22). In addition, we observed that the abundance of viral structural genes was significantly lower at 45 to 55 cm than at 15 to 25 cm (paired *t* test, *P < *0.05). This result was consistent with the differences of viral genomic features (Fig. 1B and C), further indicating that soil depths could be an important factor in shaping the evolution of viral genomes. However, neither warming nor soil depths had significant effects on the abundance of viral genes related to replication or infection (Fig. 5B).

### Virus-microbe lineage analysis.

A total of 201 microbial metagenome-assembled genomes (MAGs) were binned from metagenomes, including 188 bacterial and 13 archaeal MAGs spanned 12 bacterial and 2 archaeal phyla (see Fig. S6). After clustering, 88 microbial operational taxonomic units (mOTUs) were used for subsequent analysis. The mOTUs were highly represented by the phyla *Actinobacteria* (*n* = 21), *Proteobacteria* (*n* = 14), “*Candidatus* Dormibacteraeota” (*n* = 12), and *Acidobacteria* (*n* = 11). Based on the normalized abundance among the samples, microbial communities had more similar abundance patterns at the same depth, while these highly represented phyla was highly abundant at both two depths (see Fig. S6). Several methods were then used to predict viral hosts and to establish links between the vOTUs and mOTUs. Sequence homology and CRISPR spacers are the most effective methods to identify viral hosts (35), and previous studies have pointed out that viruses can obtain tRNAs from their host genomes during the process of infection (36). These methods for finding signals for the exchange of genetic material between vOTUs and mOTUs provided host information for approximately 19.4% of vOTUs with the oligonucleotide frequency (ONF) method identifying possible hosts of another 2.3% of the vOTUs (see Table S2).

10.1128/mbio.03009-22.6 FIG S6 Abundance patterns of mOTUs across different samples. Samples are clustered based on the Bray-Curtis dissimilarity. The color intensity in each panel represents the normalized abundance of mOTUs of each lineage. Download FIG S6, TIF file, 0.2 MB. Copyright © 2023 Ji et al. 2023 Ji et al. https://creativecommons.org/licenses/by/4.0/ This content is distributed under the terms of the Creative Commons Attribution 4.0 International license.

10.1128/mbio.03009-22.9 TABLE S2 Host prediction of 1,385 viral operational taxonomic units. Download Table S2, XLSX file, 0.1 MB. Copyright © 2023 Ji et al. 2023 Ji et al. https://creativecommons.org/licenses/by/4.0/ This content is distributed under the terms of the Creative Commons Attribution 4.0 International license.

**FIG 6:**
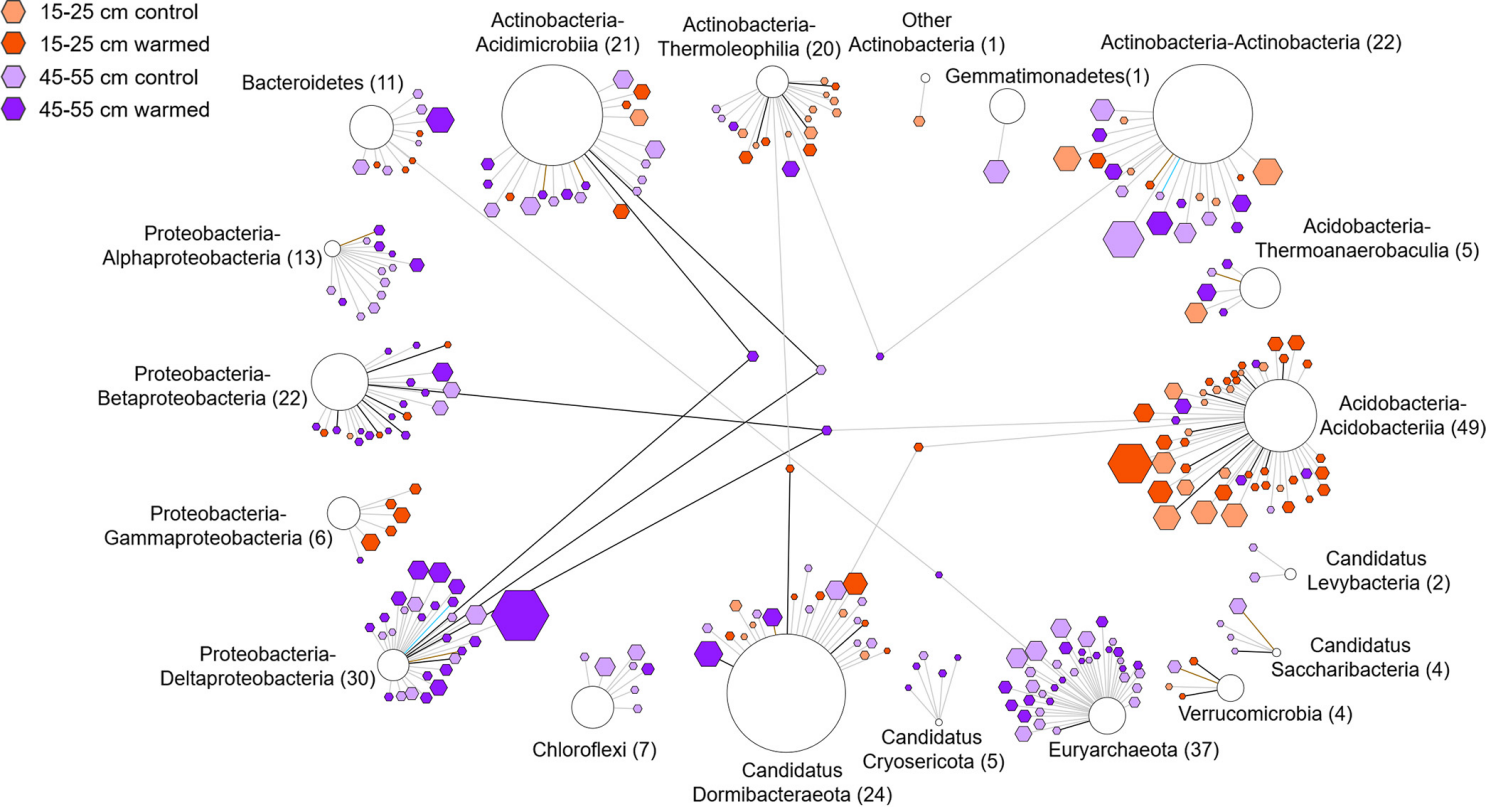
Virus-host interaction network. Viral genomes (hexagons) are connected to predicted microbial host genomes (circles) by edges. Viral genomes are sized by abundance (normalized mean coverage) across data sets. Host genomes are sized by all mOTUs abundance (normalized mean coverage) that are assigned to vOTUs across data sets. For the same host predicted by multiple pipelines, the edge colors are visualized in the following order: (i) CRISPR spacers, (ii) tRNA, (iii) BLASTn, and (iv) ONF. Edges between viral and host genomes are colored based on prediction sources: CIRSPR (cyan), tRNA (black), ONF (brown), and BLASTn (gray). The numbers in the graph represent the degree of connectivity to nodes.

At the phylum level, the host range of these viruses (276 of 1,385 vOTUs) covered 12 bacterial and archaeal phyla (excluding the phyla *Thaumarchaeota* and “*Candidatus* Aminicenantes”) in our data sets (see Table S2). We then established the network analysis between 276 vOTUs and their corresponding mOTUs as previously described (Fig. 6) (37). Among them, *Actinobacteria* (~22.8%) and *Proteobacteria* (~25.4%) were the most frequently predicted host phyla (Fig. 6). At 15 to 25 cm, viruses mainly infected the phyla *Acidobacteria* (mainly including the class *Acidobacteriia*) and *Actinobacteria* (mainly including the classes *Actinobacteria* and *Thermoleophilia*), which were the most abundant in the microbial community (Fig. 6). At 45 to 55 cm, viruses infected the phyla *Proteobacteria* (mainly including the class Deltaproteobacteria) and *Euryarchaeota* (only including the class *Methanomicrobia*) were dominant, which might be related to sulfate reduction and methanogenesis (Fig. 6). In addition, we also found some viruses infected the genus *Bradyrhizobium* (belonging to the class *Alphaproteobacteria*) that potentially affected the N cycle in the soil (see Table S2). Under the experimental warming, the number of vOTUs infecting the class *Acidobacteriia* increased at 15 to 25 cm, while the number of vOTUs infecting the classes Deltaproteobacteria and *Betaproteobacteria* increased at 45 to 55 cm (Fig. 6). Warming had no effect on the number of vOTUs infecting the phylum *Euryarchaeota*. In our data sets, most vOTUs infected a specific bacterial phylum or class (Fig. 6). However, at the species classification level, there existed some vOTUs that could infect multiple mOTUs (see Fig. S7A; see also Table S2). Remarkably, the CAZymes and some genes related to S metabolism carried by viruses might not be acquired from a limited number of potential hosts, rather as a large number of different bacterial or archaeal phyla (including the phyla *Proteobacteria*, *Acidobacteria*, *Actinobacteria*, *Chloroflexi*, *Euryarchaeota*, and “*Candidatus* Dormibacteraeota”) were infected by these viruses (see Fig. S7A).

10.1128/mbio.03009-22.7 FIG S7 Analysis of virus-microbe interaction in permafrost region. (A) Virus-host interaction network at the species level. Viral genomes (hexagons) are connected to predicted microbial host genomes (circles) by edges. Viral genomes and host genomes are sized by abundance (normalized mean coverage) across data sets. Host genomes are colored according to their taxonomy. Viral genomes encoding CAZymes or AMGs for S metabolism are outlined in red and gold, respectively. The edges between the key microorganisms of the C, N, and S cycles and their corresponding viruses are colored red, brown, and purple, respectively. (B) Correlation analysis of virus-microbe abundance of each specific lineage. Linear regression model analysis is performed based on the virus-microbe abundance correlations for the specific lineage in each dataset. *R*^2^ values and Pearson’s correlations in linear regression models are presented in Table S3. Download FIG S7, TIF file, 1.9 MB. Copyright © 2023 Ji et al. 2023 Ji et al. https://creativecommons.org/licenses/by/4.0/ This content is distributed under the terms of the Creative Commons Attribution 4.0 International license.

**FIG 7:**
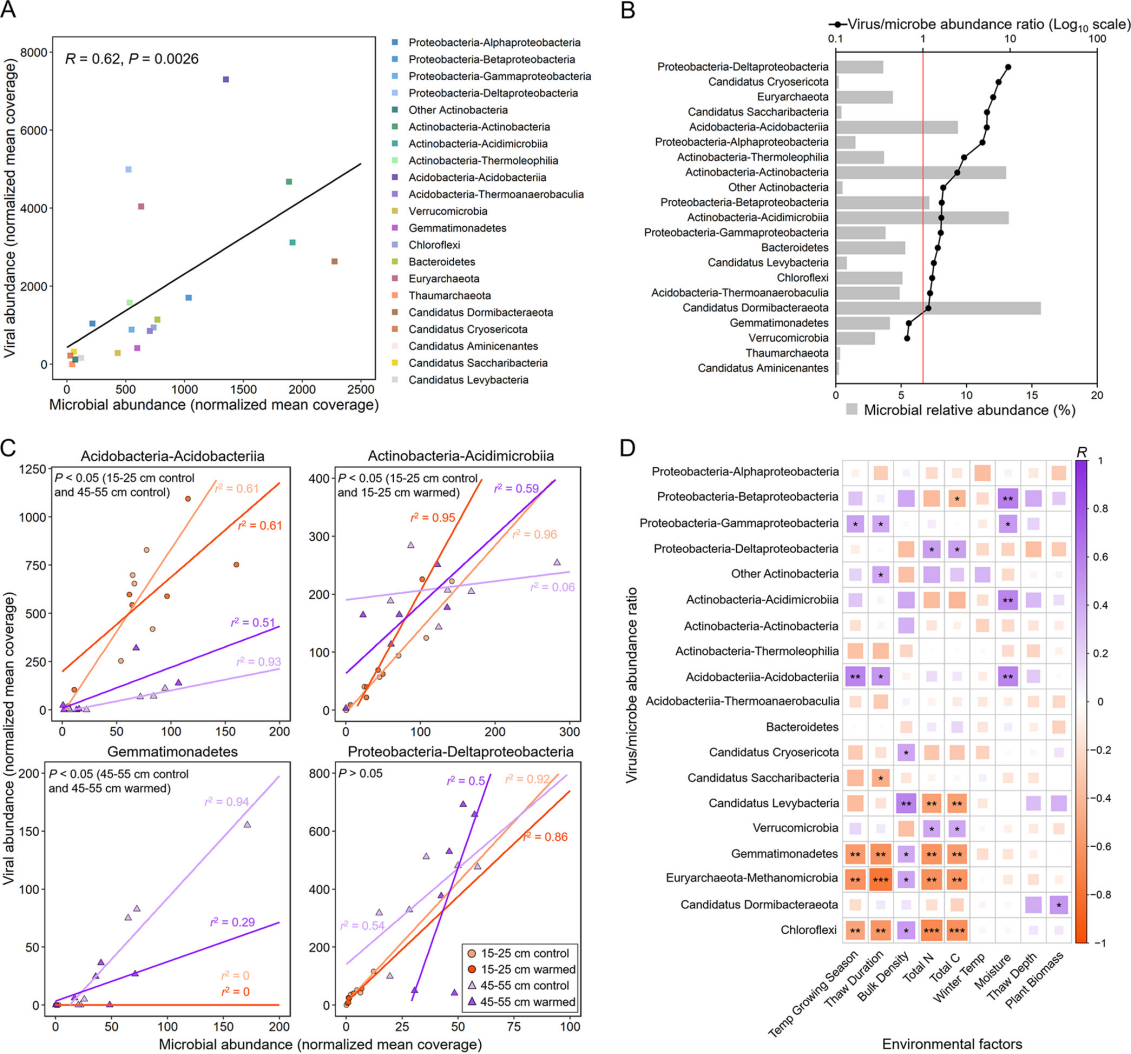
Abundance linkage between tundra soil viruses and microbes. (A) Correlation analysis between the abundance (normalized mean coverage) of microbes and their viruses. (B) VMR by virus-microbe specific lineage. The bar graph represents the relative abundance of microbes in different data sets, the dot (black) represents the VMR, and the red vertical line represents the 1:1 ratio. (C) Linear regression model analysis is performed based on the virus-microbe abundance correlations for the specific lineage in each data set. The *R*^2^ and *P* values in linear regression models are listed in Table S3. (D) Pairwise comparisons between VMRs and environmental variables. The color intensity in each panel denotes Pearson’s correlation coefficients, and the asterisks indicate Pearson’s statistical significance test results (*, *P < *0.05; **, *P < *0.01; ***, *P < *0.001).

The normalized abundance determined by reads mapping showed a strong correlation between viruses and prokaryotic microbes in permafrost region (Fig. 7A). In the virus-microbe linkage, the virus/microbe abundance ratios (VMRs) were greater than one for most specific lineages with the class Deltaproteobacteria being the highest at 9.5, indicating a higher percentage of Deltaproteobacteria population were infected by viruses (Fig. 7B). In addition, the VMRs of the phylum *Euryarchaeota* and the classes *Acidobacteria* and *Candidatus Saccharibacteria* were all greater than 5, representing the most active lineages of virus-microbe interactions in permafrost region (Fig. 7B). Analysis of virus-microbe abundance correlations for specific lineages revealed that different virus-microbe lineages had different trends in abundance changes with sustained experimental warming (Fig. 7C). Meanwhile, the specific virus-microbe lineages responded differently to warming at the two depths (Fig. 7C; see also Table S3). Under warming, the VMRs of the class *Acidimicrobia* increased significantly (two-way analysis of variance [ANOVA], *P < *0.05) at 15 to 25 cm, while the VMRs of the phyla *Verrucomicrobia*, *Candidatus Cryosericota*, and *Gemmatimonadetes* decreased significantly (two-way ANOVA, *P < *0.05) at 45 to 55 cm (Fig. 7C; see also Fig. S7B and Table S3). This indicated that the viral lytic capacity of these bacteria was altered under sustained warming. In addition, we observed that the abundance relationships of a large number of virus-microbe lineages (including the classes *Gammaproteobacteria*, *Acidobacteriia*, etc.) had significant differences between the different depths (two-way ANOVA, *P < *0.05), indicating that the infection mode of soil viruses was related to depth (Fig. 7C; see also Fig. S7B and Table S3). The linear mixed-effects models were performed for the VMR of each specific lineage that was significantly affected by both warming and depth. The results showed that soil depth not warming was a more important factor influencing the VMRs of *Verrucomicrobia* and *Acidimicrobiia* (see Table S3). Interestingly, the VMRs of some virus-microbe lineages were also significantly correlated with some environmental factors (Fig. 7D). For example, the VMRs of the two usually competing groups in soil habitats, the class *Methanomicrobia* and the class Deltaproteobacteria, were negatively (*P < *0.01) and positively (*P < *0.05) correlated with total C, respectively (Fig. 7D). This indicated that viruses may regulate the dynamics of microbial communities in response to changes in the surrounding nutrients.

10.1128/mbio.03009-22.10 TABLE S3 Linear mixed-effects model analysis and linear regressions of the normalized abundance of the microbes and their viruses. Download Table S3, XLSX file, 0.01 MB. Copyright © 2023 Ji et al. 2023 Ji et al. https://creativecommons.org/licenses/by/4.0/ This content is distributed under the terms of the Creative Commons Attribution 4.0 International license.

## DISCUSSION

Compared to marine viral communities, the functional roles that viruses play in soil ecosystems is poorly understood. This is largely due to the complex structure of soils (38). With the advancement of experimental and sequencing technology, several studies on soil viruses have been conducted, involving mangrove, tundra, farmland, and other soil ecosystems (15, 39, 40). While the response of soil microorganisms induced by climate warming has received extensive attention, only a few studies have focused on the response of viral communities (15, 25). The present study explored the response of viral communities to warming and other environmental factors and compared the differences in viral communities at two critical depths of permafrost. A total of 1,385 vOTUs were recovered, expanding the current uncultivated database (IMG/VR v4) of viruses in permafrost by 1.3-fold (41). *Caudoviricetes* was the dominant viral group in permafrost (Fig. 3C), which was consistent with several other soil ecosystems such as mangrove and farmland (39, 40), showing that *Caudoviricetes* can largely represent the known diversity of double-stranded DNA viral distribution in soil.

Environmental conditions are major drivers of the soil microbial community development (42, 43). Viruses shape microbial community and associated processes in soil (42), but it is not known whether viruses are also driven by environmental conditions (33). Our study showed that multiple environmental factors collectively drive viral community and functional gene structures in soil above permafrost (Fig. 2B), filling major knowledge gaps in the understanding of the linkage between viruses and edaphic conditions (44). Markedly, soil depth, rather than warming treatment, was a strong factor in determining viral community and functional gene structures, which was consistent with previous study of bacterial communities in this region (23). The beta diversity, the abundance patterns of vOTUs, and the similarity clustering network based on viral shared protein clusters revealed that viral community composition was highly endemic by soil depth. Furthermore, the abundance of some viral functional gene classes and specific virus-microbe lineages was also strongly stratified.

The long-term experimental warming did not alter the composition of viral communities at two depths, but affected viral functional potential related to AMGs. This was similar to some previous studies on microorganisms (45), showing that functional potential was more sensitive to warming than community composition. We believed that tundra soil viruses were sensitive to warming, but the viral evolution driven by warming in a short period of time was only manifested in changes in viral functional traits rather than viral community. However, some significant changes in microbial community composition of tundra soil under warming were observed at the CiPEHR site (2, 10), suggesting that viral communities may be less sensitive to warming than bacterial and fungal communities. Remarkably, AMGs encoded by viruses related to C metabolism were relatively abundant, especially at 15 to 25 cm warmed (Fig. 4). Recently, some virus-encoded GHs with precise functions have been identified in peatland and mangrove soils. Although their specific mechanisms were unknown, it is certain that they played important roles in the degradation of complex carbohydrates (15, 39). A total of 11 GH genes belonging to 9 families were identified in 10 vOTUs, most of which (10 of 11 GHs) were identified at warming groups (Fig. 4B). Interestingly, 6 of 10 vOTUs carrying GHs were lysogenic viruses (see Fig. S3A). These lysogenic viruses could acquire the ability to degrade SOC from their hosts by HGT and then pass on this ability when infecting other hosts, which might be another critical way for viruses to regulate the soil C cycle (29, 46). In the process of virus-host coevolution, the frequency of HGT is extremely high (47). Climate warming has been shown to drive the evolution of microbial networks in soil toward greater complexity and stability (48, 49). In this case, the acquisition of essential functional genes through HGT was the result of the coevolution of the entire microbial ecological network (50, 51).

Several recent studies focusing on the involvement of viruses in the S cycle have found that they could encode AMGs related to S metabolism that affect this biogeochemical cycle (28, 52). The findings in sulfidic mine tailings indicated that viruses could help their hosts to utilize sulfate under anoxic conditions and thereby facilitate their own reproduction and metabolism (28). Combined with the viral feedback in tundra soil, we believed that warming might promote the reduction in redox potentials, thus allowing some viruses to carry AMGs related to assimilatory sulfate reduction pathway at 45 to 55 cm. In addition, we also observed a reduction of AMGs related to organic S transformation carried by viruses at 45 to 55 cm under warming (Fig. 4B). These results suggested that viruses tended to participate in inorganic S transformation (especially sulfate reduction) under warming, which might be beneficial to viral survival. As an important process for microorganisms to participate in the S cycle, sulfate reduction also plays a key driving role in the soil C cycle (53). We speculated that warming might promote viruses to participate in the soil S cycle, mainly in the process of sulfate reduction. Occurring simultaneously, the regulation of S cycle by viruses might affect the soil C cycle, which is mainly reflected by promoting the loss of SOC, ultimately aggravating climate warming (53).

The study of tundra soil microbial metabolism showed that the relative abundance based on 16S rRNA gene sequence of *Euryarchaeota* at 45 to 55 cm in this permafrost region was significantly increased under experimental warming (2). Correspondingly, a large number of vOTUs that infected *Euryarchaeota* were mined in the 45- to 55-cm data set. As methanogens (particularly those using acetate for CH_4_ production) were stimulated by warming, the content of total C in soil was significantly reduced (*P < *0.05). This might lead to more viral infection of methanogens (belonging to the class *Methanomicrobia*) and less viral infection of sulfate-reducing bacteria (belonging to the class Deltaproteobacteria), thus allowing the sulfate reduction pathway to dominate at 45 to 55 cm (Fig. 7D). The regulation of the dynamic balance of the two usually competing groups by viruses might facilitate the mitigation of methane production. In addition, a previous study showed a greater response of methanogenesis from acetate compared to other methanogenesis pathways under warming, mainly due to the plant-microbe interactions caused by the increased biomass of *Eriophorum vaginatum* and the prevalence of *Carex rostrata* (2). Through the virus-microbe linkage, we believed that the regulation of hosts by viruses might be another key factor causing the response of methanogenesis. Warming and thawing might change the predation strategy of viruses on some methanogens, which promote a shift in the type of methane production from mostly hydrogenotrophic to more acetoclastic (7, 15). In conclusion, these results demonstrated the potential contribution of viral infection and lysis to the biogeochemical cycling in tundra soil ecosystems under sustained warming and thawing.

Viral ecology studies showed that encoding AMGs and lysing microbes to release C were the two main pathways for viruses to affect biogeochemical cycles (54). However, the extent to which the two pathways contribute to the C cycle is not yet clear. In our research, the viral life cycle strategies and viral infection patterns were significantly different at two depths of this permafrost region (Fig. 5; see also Fig. S4). At 15 to 25 cm, a higher proportion of lysogenic viruses facilitated the occurrence of HGT (46), allowing more viruses to carry GHs to participate in the C cycle. At 45 to 55 cm, some viruses tended to encode more abundant genes related to lysis function, releasing C that fueled the microbial food web through the stronger viral shunt. Considering the more rapid feedback of microbial communities to warming at 45 to 55 cm indicated by previous studies (2), we speculated that the contribution of viral shunt to the C cycle might be higher than that of encoded AMGs over a short period of time. The differences in the main ways of viruses to influencing the C cycle at might be an important factor for the different microbial feedbacks to warming at the two depths.

Lysis-lysogeny decision-making in viruses may be determined by surrounding nutrients and microbial population density based on previous studies (14, 55). Normally, the VMRs were correlated positively with microbial population density when they increased sufficiently high, which was consistent with the “kill the winner” hypothesis (56, 57). A recent study on vertical gradients of soil viruses showed that the abundance of viruses in agriculture soil area decreased (42) and lysogeny increased with increasing soil depths (34). However, our study indicated that tundra soil viruses had different patterns. Compared to the transition zone at the permafrost-active layer boundary (45 to 55 cm), the active layer (15 to 25 cm) had greater nutrient abundance (such as total C and N) and microbial metabolic genes (2). However, we observed fewer viral members and a higher proportion of lysogenic viruses at 15 to 25 cm than at 45 to 55 cm (Fig. 1A and Fig. 5A). Although these results are contrary to our conventional knowledge, they can be well explained by the “Piggyback-the-Winner” hypothesis (58). This hypothesis suggests that more viruses select for lysogeny when microbes are rapidly growing and performing active metabolism, thus conferring competitive advantage to commensals against niche invasion (58, 59). Several viral studies in coral reefs (59) and mammalian gut ecosystems (60) have supported this hypothesis. Therefore, “Piggyback-the-Winner” may be a widely adopted strategy for tundra soil viruses, although the factors driving this pattern are not clear. Future efforts are needed to verify the “Piggyback-the-Winner” hypothesis in tundra soil viruses and to resolve the mechanisms shaping viral life cycle strategies.

### Conclusions.

Here, we systemically explored the composition and function of viral communities at two soil depths in response to warming in Alaskan tundra soils. The results showed that viral functions were sensitive to warming, which was mainly reflected in the increased number of GHs encoded by viruses, and the altered VMRs for specific lineages. Environmental factors were major drivers shaping viral community and functional gene structures, and soil depth had a greater impact on viral community and functional composition than experimental warming. Furthermore, the strong viral shunt and the altered predation strategies of viruses on microbes might be relevant to the greater abundance of genes related to microbial C metabolism at 45 to 55 cm. Also, the HGT mediated by lysogenic viruses at 15 to 25 cm might make an important contribution to the increase in abundance of GHs. In addition to the effects on the soil C cycle, some viruses were found to regulate other biogeochemical cycles by encoding genes related to S metabolism and infecting some key microorganisms of the S and N cycles. In a word, our research showed that viruses contribute to the response of microbial communities to warming via diverse ways and highlighted viral importance in soil ecological research. However, considering that metagenomic analysis can only recover a very small portion of viral communities, and is less reproducible and quantitative (21, 61), a comprehensive study using a combination of viromes, metagenomes, and GeoChip is needed to better understand the responses of viral communities and associated functional genes to warming.

## MATERIALS AND METHODS

### Data acquisition and *de novo* assembly.

All high-throughput sequencing reads used in this research were obtained from NCBI Sequence Read Archive (SRA) database, derived from a previously northern-latitude tundra soil microbiome study (2). Here, two critical permafrost depths: 15 to 25 cm (active layer at outset of the experiment) and 45 to 55 cm (transition zone at the permafrost-active layer boundary at the outset of the experiment) of Alaskan tundra soils at CiPEHR site (63°52′59″N, 149°13′32″W) were warmed *in situ* (~1.1°C above ambient temperature) for five winters from 2008 to 2013. Then, soil DNA was extracted from the warmed and control groups, and sequenced on an Illumina HiSeq 2500 instrument (150 bp, paired-end mode). In addition, data were obtained for environmental factors and GeoChip at CiPEHR site with the same coordinates and sampling dates from another study (17). Here, tundra soil functional genes were analyzed with a microarray-based tool (GeoChip 5.0) containing 161,961 probes belonging to 1,447 gene families, among which there were 2,848 virus-specific probes (17, 22). Raw signal intensities after treating and analysis were normalized by Feng et al. (17).

Raw sequencing reads for all samples (15 to 25 cm, control [*n* = 6]; 15 to 25 cm, warmed [*n* = 6]; 45 to 55 cm, control [*n* = 6]; 45 to 55 cm, warmed [*n* = 6]) were trimmed using CLC Genomic Workbench (v20.0.3; Qiagen Bioinformatics, Denmark) with the following parameters: quality score limit, 0.05; maximum number of ambiguities, set to 2; and minimum read length, 35 bases (62). Next, filtered reads from each metagenome were separately assembled with CLC’s *de novo* assembler using the default parameters for downstream analysis.

### Metagenome-assembled genome binning and microbial OTU grouping.

For each assembly sets, contigs were binned using metaWRAP v1.3.2 with binning module (–metabat2 –maxbin2 –concoct) and Bin_refinement module (>50% completeness and <10% contamination) (63). The final produced bin sets were clustered and dereplicated at 95% average nucleotide identity (ANI) using dRep v3.2.2, generating 88 microbial operational taxonomic units (mOTUs) (64). mOTU taxonomies were assigned using GTDB-tk v1.7.0 based on the Genome Taxonomy Database R06-RS202 (65, 66). The classification results were further refined by comparing to NCBI taxonomy. Finally, the marker genes of MAGs identified by GTDB-tk were used to construct the bacterial and archaeal trees by RAxML v8.2.12 (67).

### vOTU table generation.

VirSorter v1.0.3 (68), DeepVirFinder v1.0 (69), VirSorter2 v2.2.2 (70), and CheckV v0.7.0 (71) were used to recover metagenomic viral contigs (mVCs) from the contigs assembled by CLC’s *de novo* assembler. For contigs of ≥5 kb (72, 73), mVCs were identified based on the following criteria: (i) VirSorter (virome database) categories 1, 2, 4, and 5 (for proviruses, only proviral regions were retained) (15); (ii) DeepVirFinder (default settings) score ≥ 0.9 and *P ≤ *0.01; (iii) high confidence level (score ≥ 0.9 or score ≥ 0.7 but have hallmark genes) of VirSorter2 (–keep-original-sequence); and (iv) VirSorter categories 3 and 6 (proviral regions), DeepVirFinder score ≥ 0.7 and *P < *0.05, and VirSorter2 score ≥ 0.5 were further screened using CheckV (containing at least one viral hallmark gene) (71). For contigs ≤5 kb, VirSorter2 was applied to identifying the viruses. Only the high-confidence and circular contigs were retained and further filtered by CheckV. The final data sets of mVCs recovered were extracted proviral regions according to CheckV. Next, all mVCs were dereplicated and clustered at 95% ANI and 85% alignment fraction of shorter sequences using CD-HIT v4.8.1, generating 1,385 viral operational taxonomic units (vOTUs) (74). For these vOTUs, genome completeness assessment was performed using CheckV.

### Taxonomic composition of vOTUs.

Prodigal v2.6.3 (-p meta) was used to predict open reading frames (ORFs) of the 1,385 vOTUs (75). The vOTUs were classified as previously described. To begin analysis, all proteins encoded by viruses were utilized as input files in vConTACT2 v0.9.19 to run against the NCBI Viral RefSeq v85 database and Stordalen Mire viruses (15, 76). The vOTUs were clustered based on the similarity of shared protein clusters between genomes, resulting in the construction of viral gene sharing network. Then, cytoscape v3.8.0 was used to visualize the network (77). Considering the major revisions of viral taxonomy in ICTV (78, 79), the vOTUs were further annotated using PhaGCN v2.1 with the latest ICTV classification (80, 81).

### Annotation of permafrost viral AMGs.

To determine AMGs contained by viral genomes, viral genes were identified using the following methods. (i) Proteins encoded by viruses were input to DRAM-v pipeline (82). AMGs identified by DRAM-v with unknown function were then compared to the CAZymes, NCyc, and SCyc databases using diamond (identity ≥ 40%, coverage ≥ 60%, and E value < 1E−10) and the KEGG database using KofamKOALA (E value < 1E−10, and HMM score > 50) to determine their potential function (see Table S1) (83–86). This process excluded 184 vOTUs owing to being discarded by Virsorter2 (70). (ii) Annotation of viral AMGs based on VIBRANT pipeline (see Table S1) (87). For these putative AMGs verified by DRAM-v and VIBRANT, we manually managed to select CAZymes, as well as N and S metabolism genes (see Table S1). Finally, Phyre2 was used for protein function prediction and structural modeling (see Fig. S3C) (88).

### Genomic analyses of viruses.

The functions of viral genes excluding AMGs were predicted by screening against the NCBI-nr database using BLASTp (bit score > 50, and E value < 1E–5) (89). Genome maps of viruses were carried out with Geneious (90). For the identification of lysogenic signals (including integrase, recombinase, repressor, or provirus) (52), the proteins encoded by viruses were run against EggNOG and Pfam database and were manually confirmed based on BLASTp against NCBI-nr database (bit score > 50, and E value < 1E–5) (84, 91).

### Host prediction of viruses.

Several different methods were used to assign hosts to vOTUs, including nucleotide sequence homology, CRISPR spacers matches, tRNA gene matches, and oligonucleotide frequency (ONF). First, viral sequences were homologously matched to recovered MAGs based on shared genomic regions through BLASTn (24). Only the best matches that hit ≥2,500 bp and had a ≥70% identity were retained (92). For some circular viral contigs or proviruses of ≤5 kb, the prediction criteria proposed by Li et al. was followed (29). Next, the CRISPR spacer sequences in the viral and host genomes are reliable features for identifying recent virus-host interactions as hosts place a fragment of the infecting phage sequence into CRISPR arrays on their genome (35). The CRISPR spacer sequences in MAGs were automatically identified using CRISPR CasFinder (93) and compared to viral sequences using BLASTn with the following parameters: E value threshold of 1E−5, percentage identity of 95%, using 1 as a maximum target sequence. Next, the tRNA genes contained in the viral sequences were identified by tRNAscan-SE v2.0.9 and aligned with the recovered MAGs using BLASTn (sequence identity and coverage ≥ 90%) (94). Lastly, hosts were assigned to vOTUs based on the similarity of k-mers (DNA sequences of length k) frequencies between sequences. VirHostMatcher v1.0 was run with default parameters (95). The matches with *d*_2_* values of <0.25 and the highest scores were retained. All prediction results and sources are listed in Table S2.

### Mapping reads to contigs and generating the mOTU and vOTU tables.

The filtered reads from the 24 samples were mapped to viral contigs (bamm make -t 40 -m 100M) using BamM v1.7.3 (https://github.com/Ecogenomics/BamM), and the BamM “filter” v1.7.3 was used to screen the reads mapped to viral contigs with the following parameters: identity ≥ 95% and coverage ≥ 90% (18, 96). CoverM v0.6.1 (https://github.com/wwood/CoverM) was first used to remove viral contigs that were covered by reads of ≤70% of their length and then used to calculate the average read depth of each contig across each sample (“trimmed_mean” coverage mode -exclude the top and bottom 10% coverage of each contig) (32). Finally, the normalized abundance was calculated for all vOTUs to allow comparison between different samples as follows: for each sample, the number of its reads was divided by one hundred million reads to generate a value, and then the average depth of each contig was divided by this value to obtain the normalized abundance (15). For the normalized abundance of each mOTUs, the coverage was calculated as the ratio of the length of each contig binned in an mOTU to that mOTU multiplied by the average depth of the corresponding contig and then summed. The same method was used for normalized abundance of vOTUs. The detailed information of the normalized abundances of 88 mOTUs and 1,385 vOTUs are separately listed in the mOTU and vOTU tables (https://doi.org/10.5281/zenodo.7442695).

### Statistical analyses.

The python script “get_ARSC.py” (https://github.com/faylward/pangenomics/) was used to calculate the GC contents and N/C atoms per residue side chain (N/C-ARSC) of vOTUs (19). Linear mixed-effects models were analyzed using the lme4 package in R v4.0.2 (97). In linear mixed-effects models, warming and soil depths were used as fixed effects, and the block was used as random intercept effect (23). ANOVA was used to calculated the *P* values, and the partR2 package was used to obtain the marginal *R*^2^ (the variance of each fixed effect) from the linear mixed-effects models (98). Alpha- and beta-diversity analyses of the viral community were performed using the vegan package in R v4.0.2 (99). The Bray-Curtis distance was calculated using the “vegdist” function of vegan and used for NMDS. ANOSIM was used to test the significance between different depths or temperatures presented in NMDS. Shannon index, richness, and Simpson index value differences between different depths or temperatures were tested using a Student *t* test (two tailed). The Mantel test was used to evaluate the correlation between environmental factors and viral community and functional gene structures. The Bray-Curtis dissimilarity values were used to represent the compositional variations of viral community and function. Dissimilarity between environmental factors was calculated using Euclidean distances based on soil winter temperature, growing season temperature, soil thaw duration, soil bulk density, total N content, and total C content. Canonical correspondence analysis (CCA) was calculated using the vegan package to model the major environmental factors shaping viral community and functional gene structures (99). To disentangle the relative importance of deterministic and stochastic processes in underlying viral community and function assembly, a total of 1,000 null models were analyzed based on Bray-Curtis distance according to a modified method as previously described (23, 100). The stochastic ratio was calculated for warming and control samples in each depth using taxonomic metrics in the NST package (100). The lineage-specific virus-microbe abundance modules for each phylum/class between different depths or temperatures were compared using two-way ANOVA, as previously described (15). Pearson correlations were performed using the “rcorr” function of Hmisc to assess the relationships between the VMRs and environmental variables (101).

### Data availability.

All raw reads used in this work are obtained from NCBI Sequence Read Archive (SRA) database generated from a previously published northern-latitude tundra soil microbiome study (2). Environmental factors and GeoChip 5.0 data at the same site of permafrost are obtained from a previously published diazotrophic community study (17). Detailed GeoChip data and environmental factor information, mOTU and vOTU tables, and mVC and MAG sequences used in this study are available at https://doi.org/10.5281/zenodo.7442695.
