# Supplementary figures and images for: Tundra Soil Viruses Mediate Responses of Microbial Communities to Climate Warming
Source: mBio. 2023 Feb 14;14(2):e03009-22. doi: 10.1128/mbio.03009-22 (PMC10127799; doi:10.1128/mbio.03009-22)

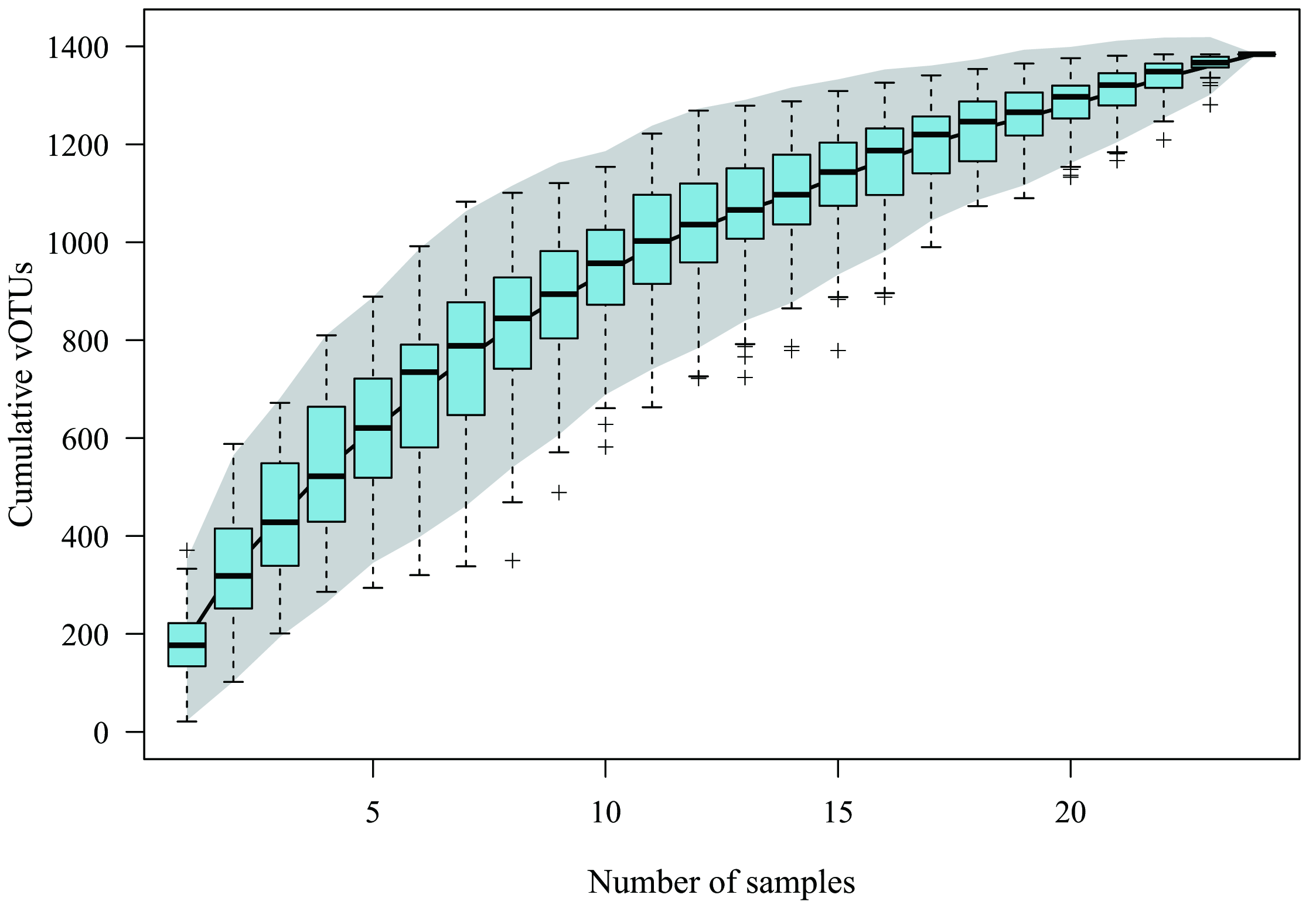

Supplement: FIG S1 [file mbio.03009-22-s0004.tif]

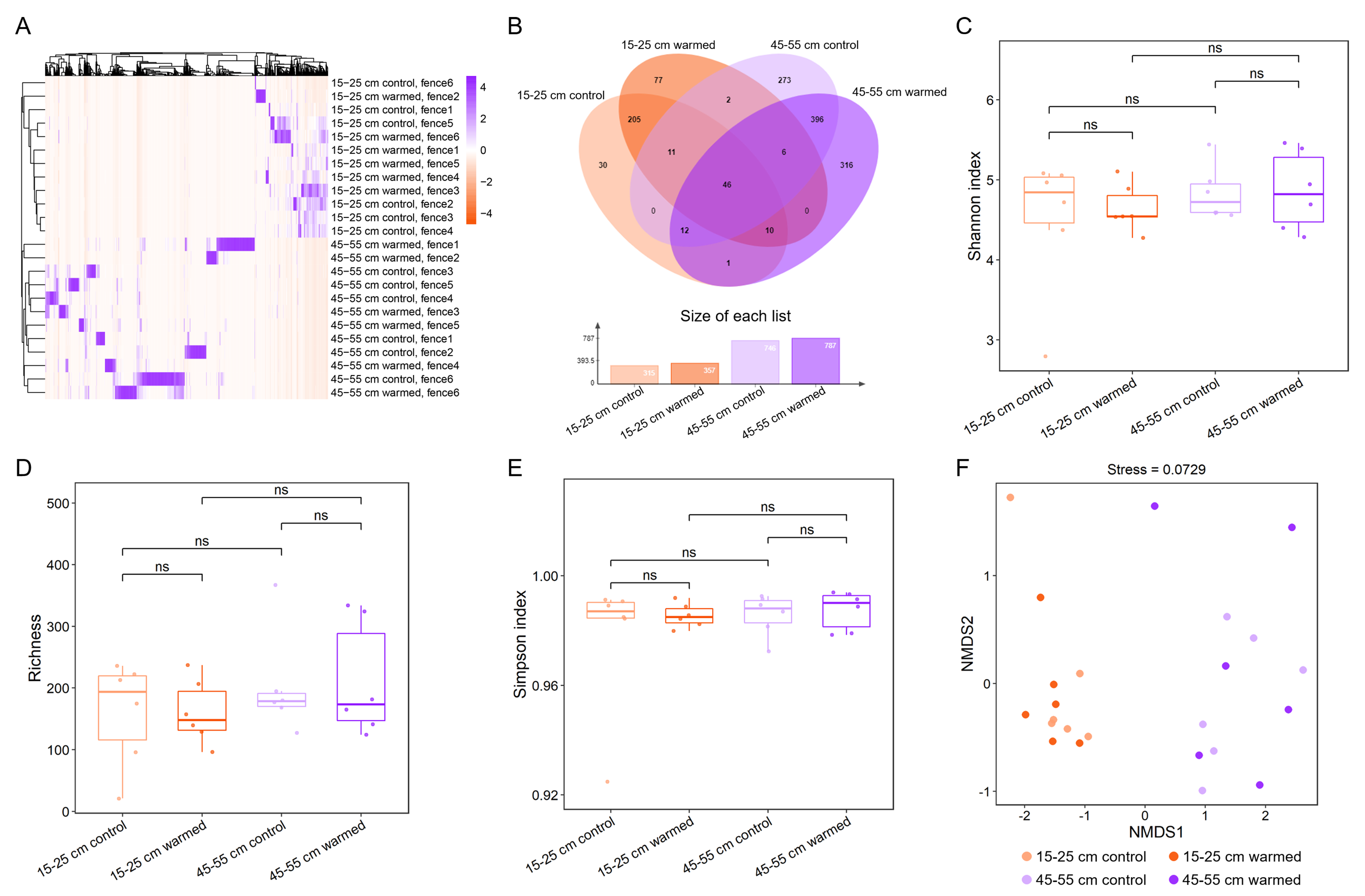

Supplement: FIG S2 [file mbio.03009-22-s0005.tif]

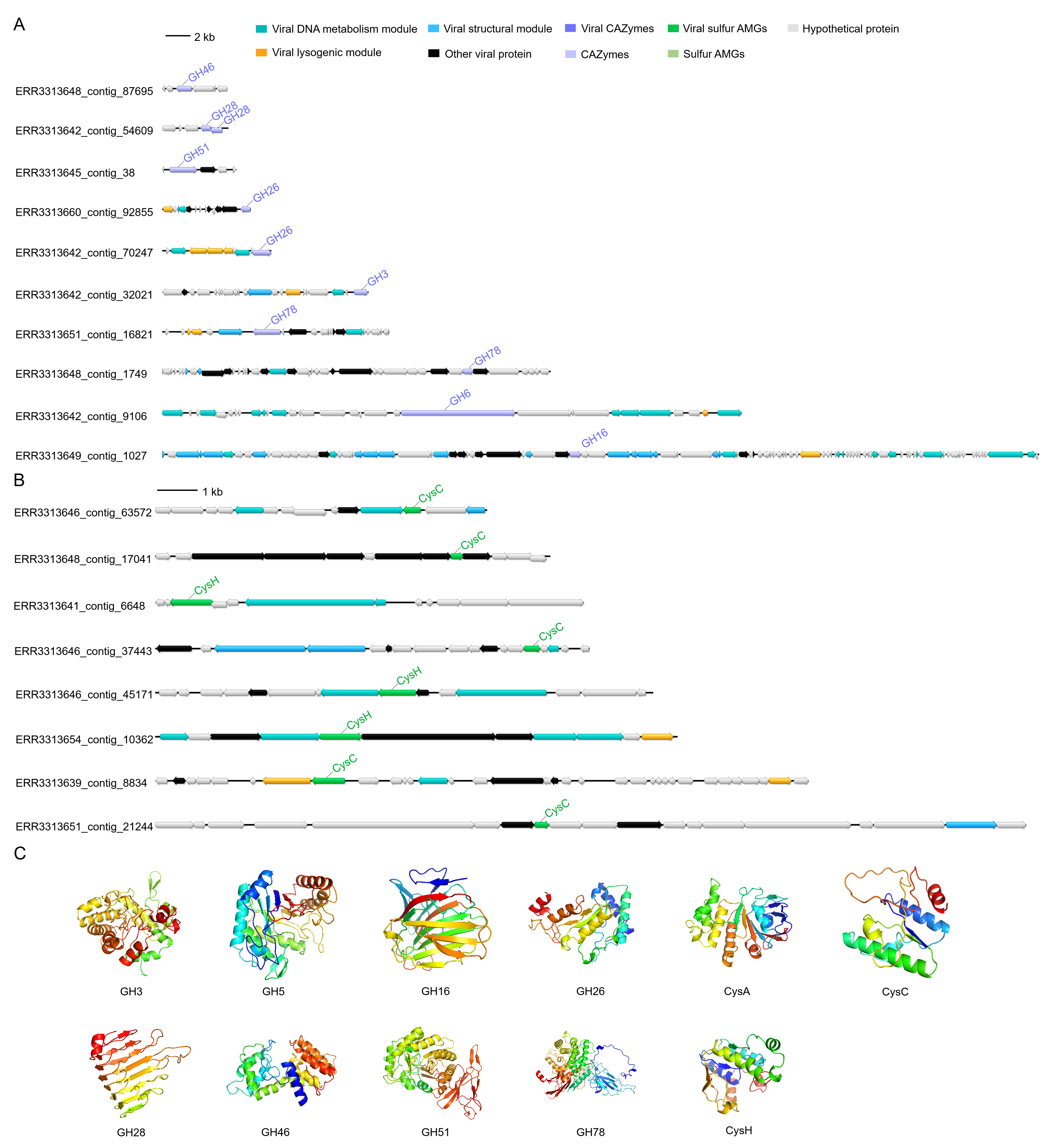

Supplement: FIG S3 [file mbio.03009-22-s0006.tif]

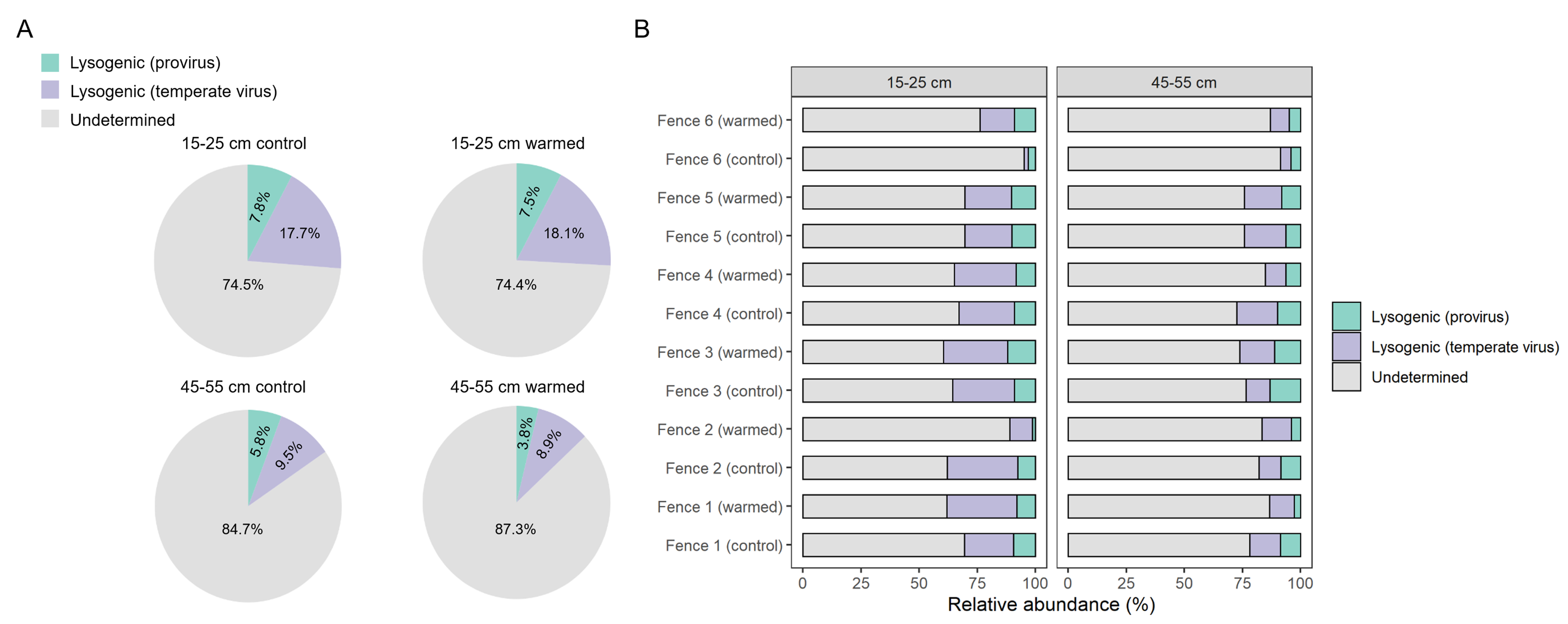

Supplement: FIG S4 [file mbio.03009-22-s0007.tif]

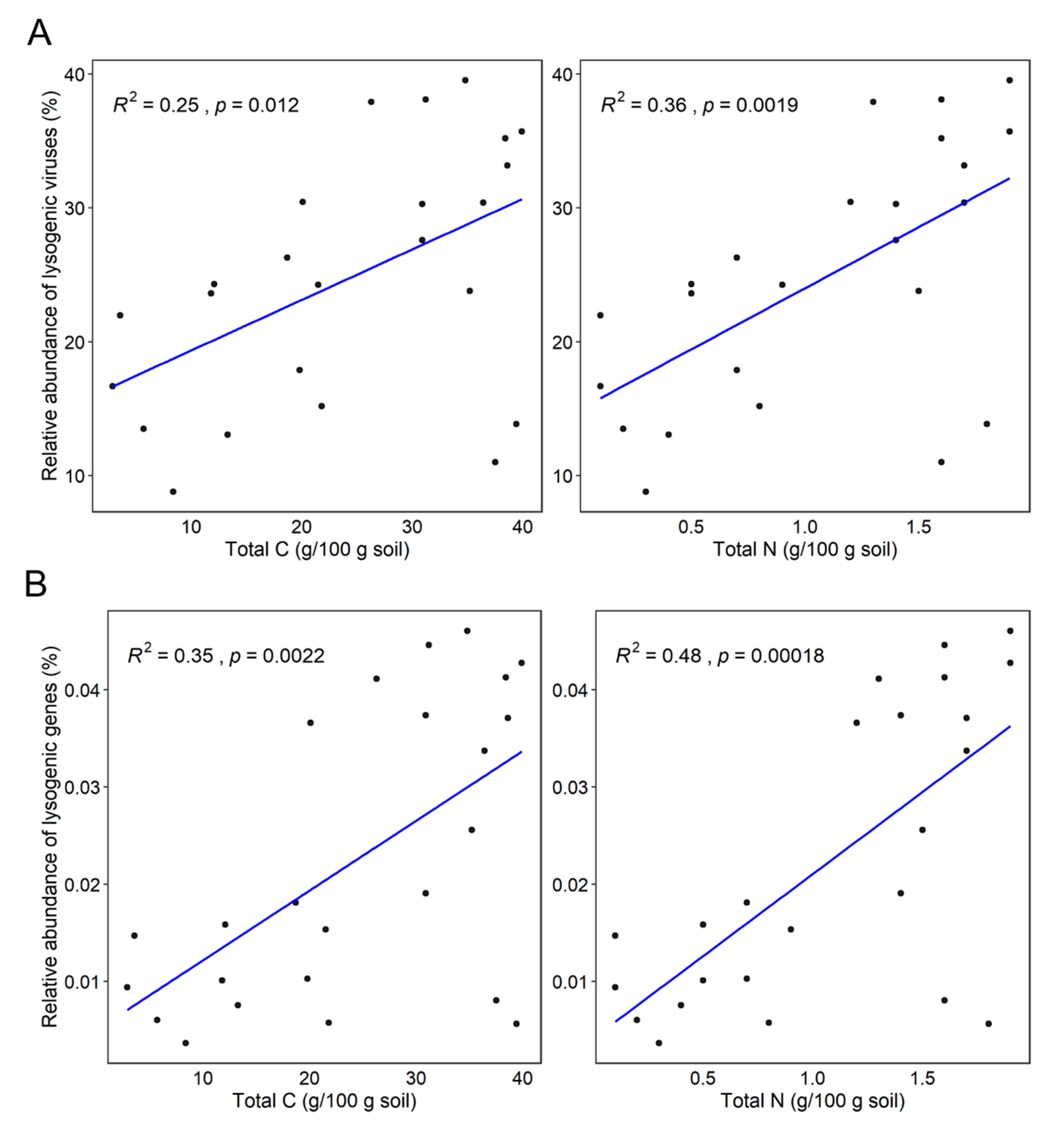

Supplement: FIG S5 [file mbio.03009-22-s0008.tif]

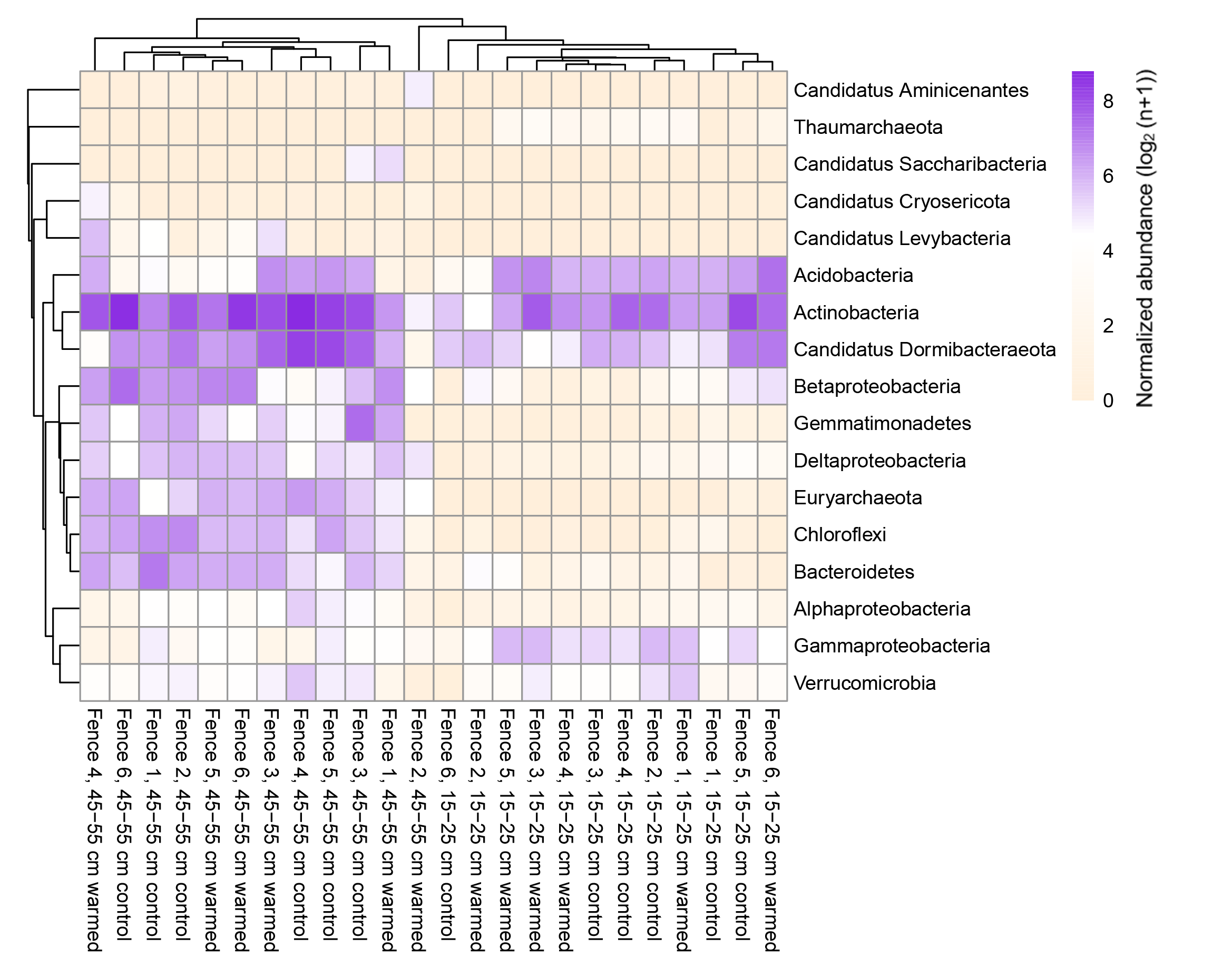

Supplement: FIG S6 [file mbio.03009-22-s0009.tif]

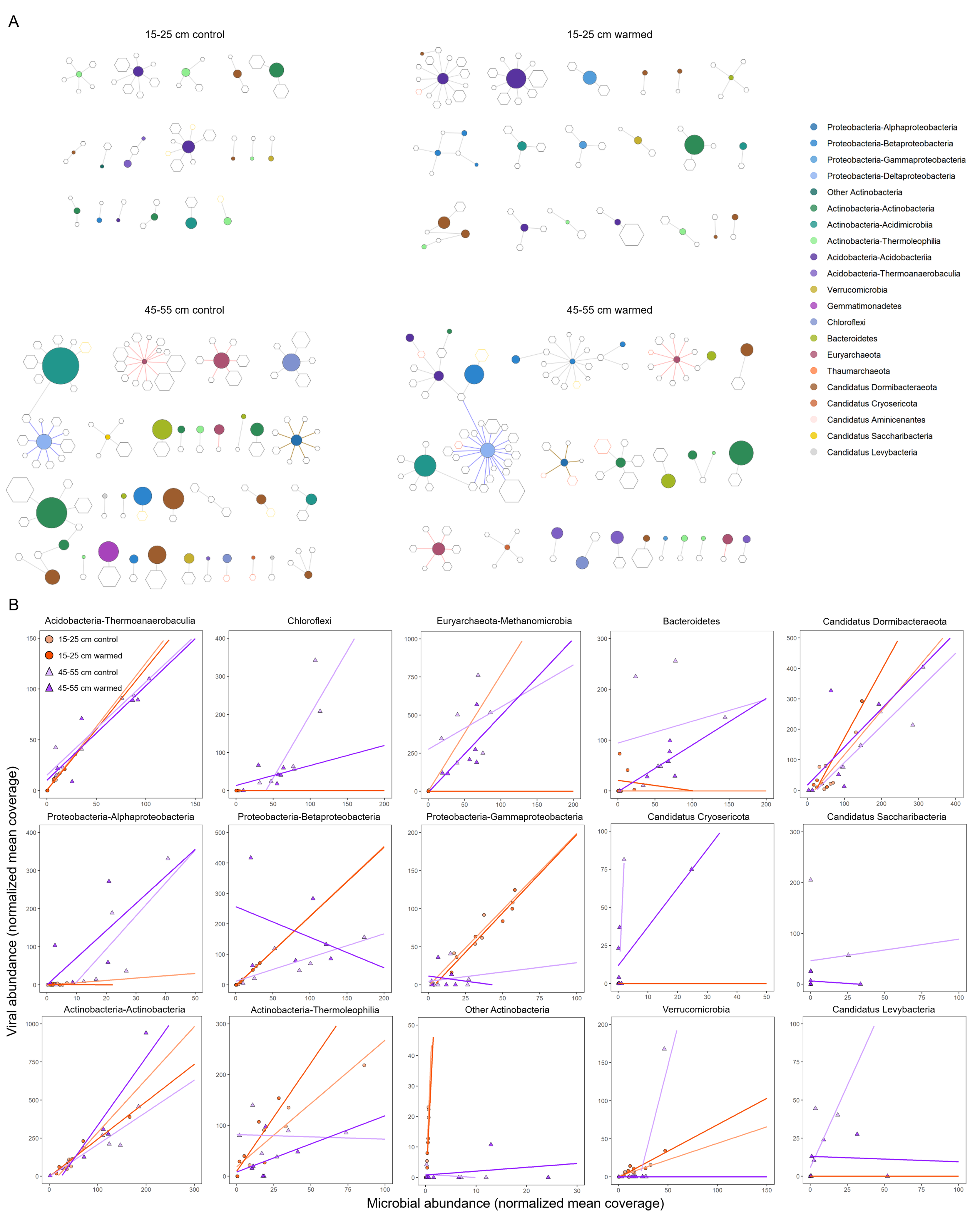

Supplement: FIG S7 [file mbio.03009-22-s0010.tif]
